# Effects of being watched on eye gaze and facial displays of typical and autistic individuals during conversation

**DOI:** 10.1177/1362361320951691

**Published:** 2020-08-27

**Authors:** Roser Cañigueral, Jamie A Ward, Antonia F de C Hamilton

**Affiliations:** 1 University College London, UK; 2 Goldsmiths, University of London, UK

**Keywords:** autism, being watched, dual function of gaze, eye gaze, facial displays

## Abstract

Communication with others relies on coordinated exchanges of social signals, such as eye gaze and facial displays. However, this can only happen when partners are able to see each other. Although previous studies report that autistic individuals have difficulties in planning eye gaze and making facial displays during conversation, evidence from real-life dyadic tasks is scarce and mixed. Across two studies, here we investigate how eye gaze and facial displays of typical and high-functioning autistic individuals are modulated by the belief in being seen and potential to show true gaze direction. Participants were recorded with an eye-tracking and video-camera system while they completed a structured Q&A task with a confederate under three social contexts: pre-recorded video, video-call and face-to-face. Typical participants gazed less to the confederate and produced more facial displays when they were being watched and when they were speaking. Contrary to our hypotheses, eye gaze and facial motion patterns in autistic participants were overall similar to the typical group. This suggests that high-functioning autistic participants are able to use eye gaze and facial displays as social signals. Future studies will need to investigate to what extent this reflects spontaneous behaviour or the use of compensation strategies.

**Lay abstract:**

When we are communicating with other people, we exchange a variety of social signals through eye gaze and facial expressions. However, coordinated exchanges of these social signals can only happen when people involved in the interaction are able to see each other. Although previous studies report that autistic individuals have difficulties in using eye gaze and facial expressions during social interactions, evidence from tasks that involve real face-to-face conversations is scarce and mixed. Here, we investigate how eye gaze and facial expressions of typical and high-functioning autistic individuals are modulated by the belief in being seen by another person, and by being in a face-to-face interaction. Participants were recorded with an eye-tracking and video-camera system while they completed a structured Q&A task with a confederate under three social contexts: pre-recorded video (no belief in being seen, no face-to-face), video-call (belief in being seen, no face-to-face) and face-to-face (belief in being seen and face-to-face). Typical participants gazed less to the confederate and made more facial expressions when they were being watched and when they were speaking. Contrary to our hypotheses, eye gaze and facial expression patterns in autistic participants were overall similar to the typical group. This suggests that high-functioning autistic participants are able to use eye gaze and facial expressions as social signals. Future studies will need to investigate to what extent this reflects spontaneous behaviour or the use of compensation strategies.

Communication with other people is based on complex exchanges of social signals, mediated by eye gaze, facial expressions, speech or gestures. Previous studies suggest that autistic individuals have difficulties in exchanging social signals, particularly via eye gaze, but evidence is mixed (Falck-Ytter & Von Hofsten, 2011). A reason for this could be that traditional experimental designs in cognitive research are not truly interactive (Redcay & Schilbach, 2019; Schilbach et al., 2013). Examining gaze patterns of autistic people in live interactions, where eye gaze has the dual function of both perceiving and signalling (Argyle & Cook, 1976; Gobel et al., 2015), could contribute towards understanding which cognitive mechanisms underlying gaze behaviour are compromised in autism. The present study addresses this question by systematically testing how ‘observing’ versus ‘interacting’ modulates gaze behaviour of typical and autistic individuals.

## Eye gaze during social interactions

Traditionally, research studying gaze behaviour has focused on how we use our eyes to perceive information from pictures and videos. Early research on visual attention showed that our eye movements are biased to attend the location in the scene that is most salient (Itti & Koch, 2001; Koch & Ullman, 1985; Veale et al., 2017). However, in social scenes, visual attention is biased towards faces and eyes of other people (Bindemann et al., 2005; Birmingham et al., 2009). This preferential bias to attend to faces likely results from the need to maximise the information we extract from others during social interactions (Cañigueral & Hamilton, 2019b; Yang et al., 2016). In line with this, Kendon (1967) proposed that during conversation our eyes monitor the attentional states and facial expressions of other people to ensure mutual understanding and approval (Efran, 1968; Efran & Broughton, 1966; Kleinke, 1986).

Recent research has used more ecologically valid designs (e.g. live interactions) to understand how we use our eyes to signal information to others. So far, findings suggest that there is little relationship between gaze patterns in computer-based tasks and gaze patterns in the real world. For instance, participants sitting in a waiting room gaze less to a live confederate also waiting in the room, than to the same confederate in a video-clip (Laidlaw et al., 2011). Participants may avert gaze from the live confederate to signal no interest in starting an interaction with a stranger (i.e. social norm of civil inattention; Foulsham et al., 2011; Goffman, 1963), or to reduce arousal associated with eye contact in live interactions (i.e. expressive function of gaze described by Kendon) (Argyle & Dean, 1965; Kendon, 1967; Pönkänen et al., 2011). This suggests that in live *non-communicative* interactions the signalling function of gaze overrides our preferential bias to attend to faces.

However, it is not yet clear how gaze patterns change from pre-recorded to live *communicative* contexts, where participants are required to actively engage with the confederate (e.g. conversation) (see Cañigueral & Hamilton, 2019a; Mansour & Kuhn, 2019 for two recent studies on this question). An important feature of communicative exchanges is that gaze patterns are coordinated with other social signals, such as speech. In a seminal study, Kendon (1967) found that transitions between speaker and listener states (i.e. turn-taking) are modulated by eye gaze, suggesting that our eyes have a regulatory function. For instance, speakers tend to avert their gaze when they begin to talk and when they hesitate (to indicate that they are going to say something), but direct their gaze to the listener when they are finishing an utterance (to indicate that the listener can take the turn) (Hessels et al., 2019; Ho et al., 2015; Kendon, 1967). On the other hand, listeners gaze at speakers most of the time to indicate interest and attention (Ho et al., 2015; Kendon, 1967). In line with this, it has been found that typical participants direct more gaze to the face of the interacting partner when they are listening versus speaking (Cañigueral & Hamilton, 2019a; Freeth & Bugembe, 2019; Mansour & Kuhn, 2019; Vabalas & Freeth, 2016; Von dem Hagen & Bright, 2017). Altogether, these findings illustrate how in live communicative interactions we plan our eye movements in relation to social signals exchanged with our partner, thus combining the perceiving and signalling functions of gaze.

## Eye gaze in autism

Autism is a neurodevelopmental condition characterised by difficulties in interpersonal interaction and communication (American Psychological Association, 2013). A hallmark of autism is abnormal gaze behaviour in infants (Zwaigenbaum et al., 2005), but evidence in autistic adults is mixed: some studies using pictures and videos as stimuli suggest that autistic adults avoid making eye contact, while others show that they have typical gaze patterns (Chita-Tegmark, 2016; Falck-Ytter & Von Hofsten, 2011; Von dem Hagen & Bright, 2017). While atypicalities in autistic gaze behaviour have been previously related to reduced *individual* interest in social interactions (social motivation theory: Chevallier et al., 2012), recent proposals suggest that social difficulties in autism result from disturbances at the *interpersonal* level, that is, in mutually attuning to each other’s signals during dynamic social exchanges (dialectical misattunement hypothesis: Bolis et al., 2018). Thus, to fully understand the cognitive mechanisms underlying social difficulties in autism, it is necessary to study gaze behaviour in *live* interactions, where gaze patterns result from the interplay of its perceiving and signalling functions.

Studies of gaze behaviour of autistic people during live interactions are scarce. To our knowledge, no study has systematically compared gaze patterns of clinically diagnosed autistic individuals in live versus pre-recorded interactions, so it is unknown to what extent they plan eye movements to signal information to others. Nonetheless, a recent study (Von dem Hagen & Bright, 2017 Experiment 1) found that, while typical participants with low autistic traits directed less gaze to a video-feed they believed to be live than to a pre-recorded video-clip, this difference was absent in typical participants with high autistic traits. This suggests that autistic individuals might not use the signalling function of gaze.

A related question is whether autistic people coordinate eye gaze with other social signals (e.g. speech) during live communicative exchanges. Only one study has looked at gaze patterns of typical versus clinically diagnosed autistic adults during conversation (Freeth & Bugembe, 2019), and two studies have compared typical adults with low versus high autistic traits (Vabalas & Freeth, 2016; Von dem Hagen & Bright, 2017 Experiment 2). Using Q&A tasks over online video-feed or face-to-face interactions, these studies report that typical/low-trait and autistic/high-trait adults follow similar gaze patterns when alternating between speaker and listener roles. However, it has also been found that adults with high autistic traits spend less time looking at a live confederate than participants with low autistic traits (regardless of speaker or listener state) (Von dem Hagen & Bright, 2017 Experiment 2), particularly at the eyes region (Hessels et al., 2018). It could be that autistic individuals find it hard to keep track of the spatio-temporal dynamics of live social interactions (Bolis et al., 2018; Cañigueral & Hamilton, 2019b): this might impose higher cognitive demands, which in turn reduces gaze directed to faces. Studying how gaze patterns of typical and autistic people develop over time and in relation to other social signals could yield further insight into which cognitive components of gaze planning are compromised in autism.

## The present study

This work investigates how typical and autistic gaze patterns are modulated by (1) the belief in being watched and (2) the potential to show true gaze direction (i.e. if actual gaze direction matches perceived gaze direction). Previous studies have focused on effects related to the belief in being watched by comparing pre-recorded videos versus video-call/face-to-face interactions, which provides a clear-cut test for the signalling function of gaze. However, little attention has been paid to the potential to show true gaze direction. This is particularly interesting in the context of video-calls, which are increasingly used in research studies to simulate live social interactions. Due to the video-camera position, in video-calls there is a mismatch between true and perceived gaze direction: this may limit the signalling function of gaze because it will be perceived as not fully coordinated with other social signals (e.g. speech). Thus, comparing video-call versus face-to-face interactions provides a manipulation of more subtle aspects of the signalling function of gaze, and can also inform about the external validity of findings obtained in video-call set-ups.

**Figure 1.**
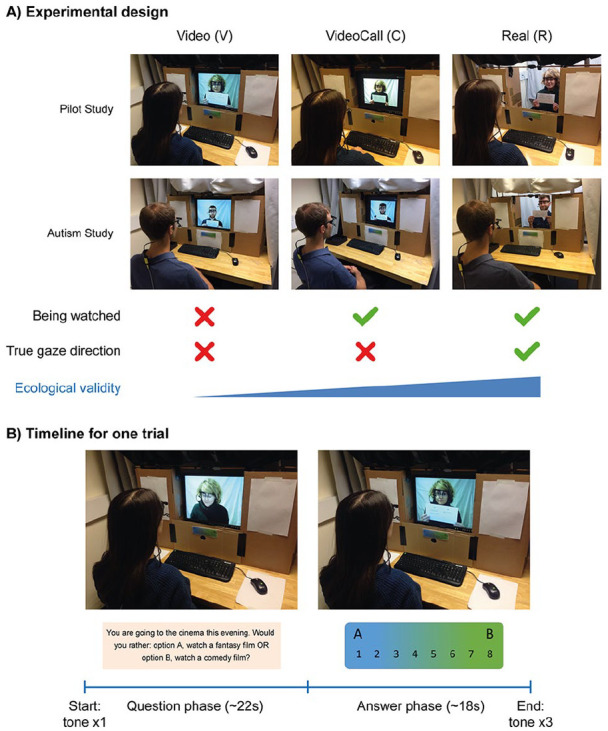
Study design: (a) experimental design and sample pictures of conditions, for both Pilot Study and Autism Study; (b) timeline for one trial in the Video condition.

Across two studies, we tested a sample of typical participants (Pilot Study; see Supplementary Materials S1), and a sample of matched typical and autistic participants (Autism Study; see present study). In each study, participants engaged in a spoken Q&A task with a confederate (professional actor) in three different social contexts: Video (pre-recorded video-clips of the confederate: gaze only has a perceiving function), VideoCall (live video-call with the confederate: gaze has perceiving and limited signalling functions) and Real (live face-to-face interaction with the confederate: gaze has perceiving and full signalling functions). These social contexts differed in the participants’ belief in being watched and potential to show true gaze direction, creating gradually increasing levels of ecological validity (Figure 1(a)).

Across all three social contexts, we recorded eye gaze of participants using wearable eye-trackers. We first analysed gaze data aggregated across the whole task for each condition. Based on our findings in the Pilot Study, we expected that typical participants would direct less gaze towards the confederate in the VideoCall and Real conditions compared to the Video condition. We predicted no differences between VideoCall and Real conditions, since our subtle manipulation for true gaze direction is probably hard to capture using aggregated measures. Moreover, if autistic individuals do not plan gaze behaviour to send signals (Von dem Hagen & Bright, 2017 Experiment 1), we should not find differences between conditions for the autistic group. We also expected that the proportion of gaze directed to the confederate would be lower in the autistic compared to the typical group for all conditions (Von dem Hagen & Bright, 2017 Experiment 2).

We then studied the dynamics of eye gaze in relation to speech across the different conditions. In line with previous studies (Freeth & Bugembe, 2019; Vabalas & Freeth, 2016; Von dem Hagen & Bright, 2017 Experiment 2), we predicted that both typical and autistic participants would direct more gaze to the confederate when they were listening than when they were speaking. Building on our findings in the Pilot Study, we also predicted that, if this effect was strictly related to regulation of turn-taking, it would only be true for the VideoCall and Real conditions. However, if cognitive demands associated with perceiving faces (Beattie, 1981; Glenberg et al., 1998; Markson & Paterson, 2009) modulate gaze planning while speaking, the effect would be true for all three conditions. The Pilot Study also revealed that typical participants gazed less to the confederate at the start and end of trials in the VideoCall and Real conditions compared to the Video condition, probably to reduce arousal. Because in the Autism study we had more accurate timing information for turns, we expected to also see a similar effect during turn-taking, likely related to the regulation of the conversation. Moreover, we predicted that autistic participants would show no such differences between conditions or over time.

Finally, we performed an exploratory analysis on participant facial motion. Previous studies have found that participants make more facial displays when they are being watched (Chovil, 1991b; Fridlund, 1991; Hietanen et al., 2019), suggesting a role for facial displays in communication (Chovil, 1991a; Crivelli & Fridlund, 2018). We tested whether typical participants move their face more when being watched, and while speaking compared to listening. A recent meta-analysis has also found that autistic participants are less likely to spontaneously produce facial displays (Trevisan et al., 2018), so we expected that the autistic group would show no differences in facial motion between conditions or time-windows, and would make fewer facial displays than the typical group.

## Materials and methods

### Participants and confederate

The Pilot Study showed a large effect size and power for the main effect of Condition on gaze directed to the Eyes of the confederate ( $n_{p}^{2}\approx 0.2$ and power ≈ 0.9 for both aggregated and time-course analyses). For the Autism Study, a power analysis indicated a required sample size of 26 participants per group (i.e. total of 52 participants) for a similar effect size and power. Thus, a group of 26 typical adults and 26 high-functioning autistic adults were recruited using the autism database at the author’s institution. Both groups were matched on age, gender, handedness and intelligence quotient (IQ; WAIS-III UK, Wechsler, 1999a, 1999b), but differed on the autism quotient (AQ; Baron-Cohen et al., 2001) (see Table 1). Specific data on ethnicity, socioeconomic status and educational attainment levels were not recorded. Note that we refer to the autistic group as high functioning, since participants’ cognitive and verbal abilities were above the typical range (i.e. IQ higher than 80). Recruitment of autistic participants was based on diagnosis from an independent clinician, either as Asperger’s Syndrome (N = 21) or Autism Spectrum Disorder (N = 5). Participants were also tested on module 4 of the Autism Diagnostic Observation Schedule (ADOS) (Lord et al., 2000) by a trained researcher: 10 participants met the ADOS classification for Autism, 10 for Autism Spectrum and 6 did not meet any classification but all 6 participants had a clear diagnosis from an independent clinician.

**Table 1.** Comparison of typical and autistic groups.

	Typical (N = 26)		Autistic (N = 26)		*t*-test	
	Mean (*SD*)	Range	Mean (*SD*)	Range	*p*-value	d_z_
Age	32.8 (10.9)	20–62	34.9 (7.71)	22–54	0.42	0.11
Gender	6 F, 20 M	–	5 F, 21 M	–	–	–
Handedness	2 L, 24 R	–	3 L, 23 R	–	–	–
IQ: full-scale	117.3 (12.0)	99–143	114.2 (11.5)	86–136	0.25	0.14
IQ: verbal	118.7 (11.7)	96–139	115.5 (10.2)	91–135	0.26	0.14
IQ: performance	112.2 (12.6)	91–140	109.9 (14.4)	80–136	0.40	0.09
AQ	13.5 (6.15)	4–28	33.1 (8.92)	10–48	<0.001***	1.32
	Meet cut off score (32): 0 participants		Meet cut off score (32): 17 participants			
ADOS: total	–	–	8.64 (3.45)	2–17	–	–
ADOS: communication	–	–	3.32 (2.39)	0–9	–	–
ADOS: social interaction	–	–	5.72 (2.44)	1–11	–	–
ADOS classification			Autism: 10 participants Autism spectrum: 10 participants Do not meet any classification: 6 participants (all have a diagnosis from an independent clinician)			

*SD*: standard deviation; d_z_: Cohen’s d for effect size; F: female; M: male; L: left; R: right; IQ: intelligence quotient; AQ: autism quotient; ADOS: Autism Diagnostic Observation Schedule.

The confederate was a professional actor (playing age: 23–29) hired for the full duration of the study to ensure a consistent performance across trials and participants. He was unaware of the aims and hypotheses of the study, and participants believed he was a student helping with the study. All participants and the confederate provided written informed consent and were compensated for their participation in the study. The study was granted ethical approval by the local Research Ethics Committee.

### Task and stimuli

Participants completed a Q&A task with the confederate. We created a set of questions for each experimental condition (Video, VideoCall and Real). Each set comprised 10 questions asking about personal preferences in neutral or prosocial daily situations (e.g. *You are going to the cinema this evening. Would you rather: option A, watch a fantasy film, or Option B, watch a comedy film?*). The three sets were matched for number of questions describing neutral or prosocial situations. See Supplementary Materials (S2) for the full list of questions used in the Autism Study.

For each trial, a single tone indicated the start of the Question phase. The confederate read a question from a card but briefly gazed to the participants’ face (webcam in the Video and VideoCall conditions) when saying ‘Option A’ and ‘Option B’ to capture the participants’ attention. After reading the question, the confederate gazed to the participant/webcam and held up the card, which had the two options written on the side visible for participants. This cued the start of the Answer phase, where participants were instructed to indicate on a scale from 1 to 8 how much they preferred that option over the other (1 = strongly prefer A; 8 = strongly prefer B), and to add further explanations about why they preferred that option. Participants spoke out their choices and explanations until they heard three consecutive tones indicating the end of the Answer phase. During the Answer phase, the confederate gazed to the participants’ face (webcam in the Video and VideoCall conditions) and displayed a polite smile. The Question phase was around 22 s long, and the Answer phase was 18 s long, so each trial had a duration of around 40 s. There was a brief rest period of 5 s between trials. See Figure 1(b) for the timeline of a sample trial.

### Experimental procedure

Participants completed the task under three experimental conditions: Video, VideoCall and Real (see Figure 1(a)). For the Video (V) condition, participants observed pre-recorded videos of the confederate while they were alone in the testing room. Participants knew the confederate could not watch them and there was no potential to show true gaze direction, resulting in a low ecologically valid interaction where gaze only has a perceiving function. For the VideoCall (C) condition, participants were alone in the room and interacted with the confederate through a freely available video-call software (Zoom). Participants knew the confederate could watch them but there was no potential to show true gaze direction (the video-camera position means there is a mismatch between true gaze direction and perceived gaze direction). This resulted in a moderate ecologically valid interaction, where gaze has perceiving and (limited) signalling functions. For the Real (R) condition, participants and confederate were in the same room, sitting across a table and facing each other. Participants knew the confederate could watch them and there was potential to show true gaze direction, resulting in a high ecologically valid interaction where gaze has perceiving and (full) signalling functions. For all conditions, the confederate was wearing a wearable eye-tracker and appeared in front of a neutral plain background.

Each experimental condition was associated with same set of questions for all participants (Set 1 – Video; Set 2 – VideoCall; Set 3 – Real). In the Pilot Study, we counterbalanced the order of the experimental conditions. Since there was no effect of order, in the Autism Study, we only used two counterbalancing conditions (*C-V-R* and *R-V-C*) that gave participants a ‘break’ between the two live interactions (less overwhelming for autistic participants). The overall duration of the study was around 45 min.

At the end of the study, all participants completed a post-test questionnaire where they indicated on a scale from 0 (disagree) to 8 (agree) how *natural* and *reciprocal* the interaction with the confederate was in each condition. Participants also indicated which interaction they liked the most and the least, and what they thought was the purpose of the experiment (responses from 11 typical and 12 autistic participants were close to the purpose of the study, for example, ‘measure eye movements when seeing a video or interacting with a real person’, but none of them guessed the meaning of our manipulation or our specific predictions). See Supplementary Materials (S3) for the full questionnaire. Afterwards, the experimenter debriefed participants about the real purpose of the study.

### Data acquisition and processing

Participants sat on one side of a table with a cardboard occluder. The occluder masked all but a 14″-squared window in front of the participant (see Figure 1). During the Video and VideoCall conditions, a 14″ monitor was fitted to the window. During the Real condition, the confederate sat on the other side of the table and his face and upper half of the body was visible to participants through the window. This set-up ensured that the confederate had similar appearance across all three conditions (see Figure 1(a)).

Two wearable eye-trackers (Pupil Core monocular, Pupil Labs, Germany) were used to record eye movements of participants and confederate. Eye-tracking data from the confederate were recorded for the VideoCall and Real conditions only, but these recordings had poor signal quality and were not used for the analyses. The Pupil Core system uses a head-mounted ‘world’ camera to record the environment, and a head-mounted ‘pupil’ camera to track the right pupil movements at a rate of 120 Hz (down-sampled to 30 Hz to match the ‘world’ camera video frame rate). In the Video and VideoCall conditions, participants sat at approximately 60 cm from the monitor and went through a 9-point screen-based calibration routine at the start of each condition (1–2 min). For the Real condition, participants sat approximately 100 cm from the confederate and went through a 6-point manual calibration routine at the start of this condition (1–2 min). After data acquisition, videos from the participants’ ‘world’ camera were further processed with OpenFace (Baltrusaitis et al., 2016) to detect facial landmark coordinates on the face of the confederate. These facial landmarks were used to create two regions of interest (ROIs) that corresponded to the upper (Eyes region) and lower (Mouth region) halves of the face, defined as the upper and lower halves of an ellipse that was adjusted to track the location and orientation of the confederate’s face during the task.

To track facial motion, a webcam was arranged to record data from the participant’s face (Logitech; recording rate of 20 Hz). Recordings were processed using the OpenFace algorithm, which uses the Facial Action Coding System (FACS; Ekman & Friesen, 1976) to taxonomise movements of facial muscles and deconstruct facial displays into specific action units (AUs). OpenFace can recognise a subset of 18 facial AUs and gives information about the presence or absence of each of these facial AUs for each frame of the video recording.

To control for facial motion effects related to speech production, two lapel microphones were used to record speech from participants and confederate. The microphones also allowed us to implement an audio trigger system to accurately detect turn-taking. The audio recordings were processed with a custom program that detected who was speaking over time. Note that there were no overall differences in the amount of participant speech across conditions (see Supplementary Materials S4).

**Figure 2.**
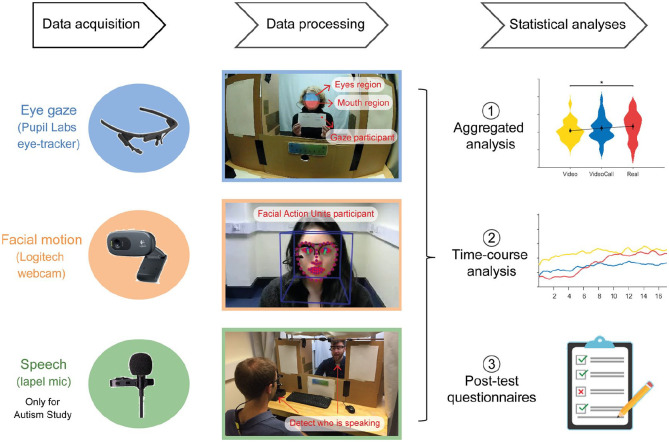
Overview of the pipeline for data acquisition, processing and analyses.

We performed aggregated and time-course analyses of eye gaze and facial motion (see ‘Results’ section for details on these analyses). See Figure 2 for a diagram with an overview of the pipeline for data acquisition, processing and analyses.

## Results

### Manipulation check: post-test questionnaire ratings

To check whether our experimental manipulation was effective, for each trait in the post-test questionnaire (naturalness and reciprocity) we run a 2-way repeated measures analysis of variance (ANOVA) with mean rating as dependent variable, Condition as within-subject factor and Group as between-subject factor. Table 2 gives descriptives for post-test questionnaire ratings.

**Table 2.** Descriptives for post-test questionnaire ratings in Autism Study.

Condition	Natural		Reciprocal	
	Typical	Autism	Typical	Autism
Video	*M* = 3.96 *SD* = 1.95	*M* = 4.58 *SD* = 1.88	*M* = 2.81 *SD* = 2.06	*M* = 3.46 *SD* = 1.96
VideoCall	*M* = 4.11 *SD* = 1.88	*M* = 5.27 *SD* = 1.61	*M* = 3.85 *SD* = 2.01	*M* = 5.15 *SD* = 1.80
Real	*M* = 4.19 *SD* = 2.19	*M* = 5.50 *SD* = 2.06	*M* = 4.61 *SD* = 2.00	*M* = 5.50 *SD* = 1.84

*SD*: standard deviation.

Scale 0 (disagree) to 8 (agree).

**Figure 3.**
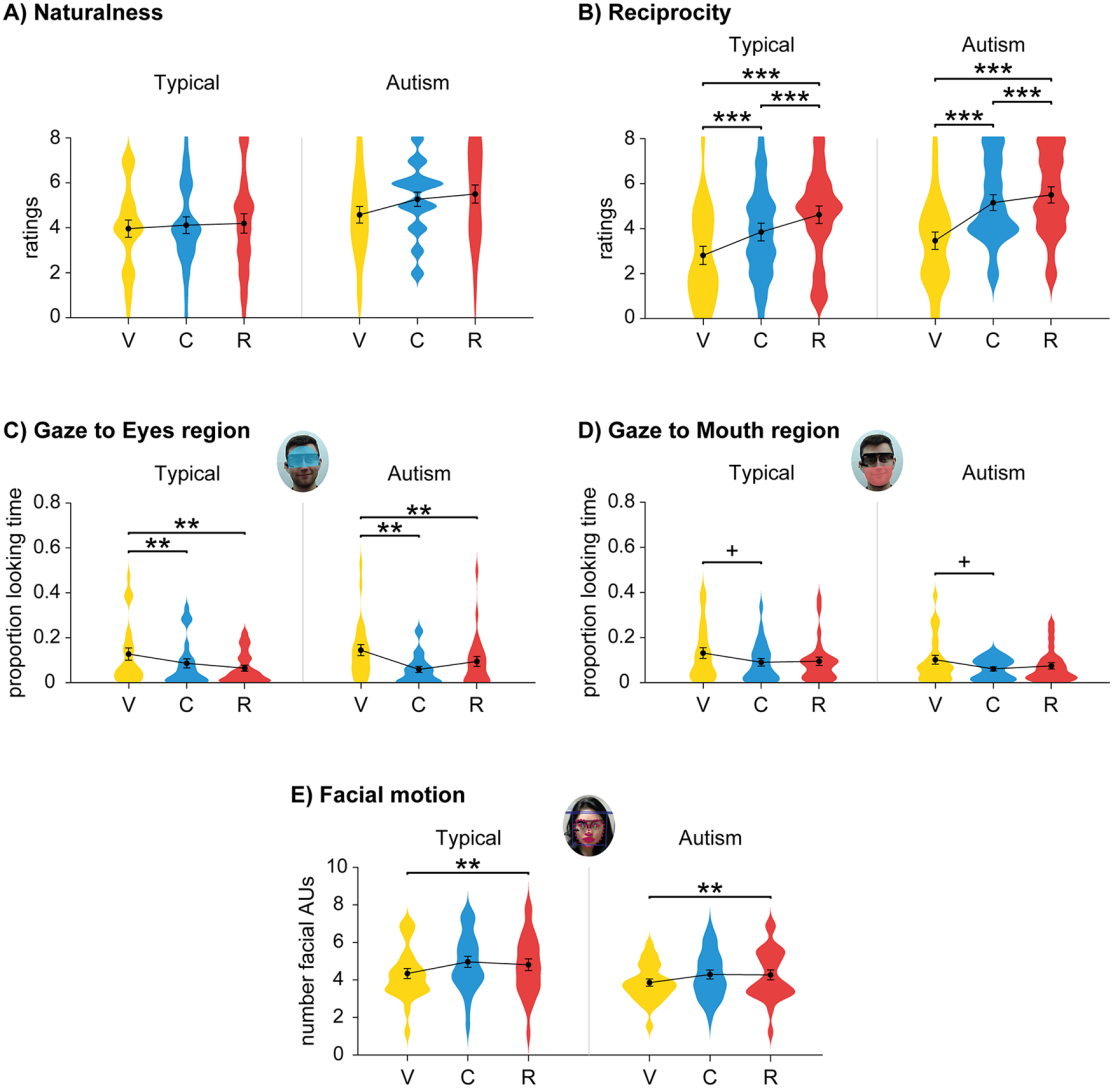
Results for ratings and aggregated analyses of eye gaze in Autism Study: (a) ratings for naturalness; (b) ratings for reciprocity; (c) proportion looking time to Eye region for each Condition and Group; (d) proportion looking time to Mouth region for each Condition and Group; and (e) number of facial AUs for each Condition and Group. Mean (filled circle), *SE* (error bars) and frequency of values (width of distribution). Asterisks signify difference at *p* < 0.05 (*), *p* < 0.01 (**) and *p* < 0.001 (***). V: Video; C: VideoCall; R: Real.

For both traits, we found a main effect of Group (naturalness: *F*(1,50) = 4.69, *p* = 0.035, $n_{p}^{2}=0.086$ ; reciprocity: *F*(1,50) = 4.03, *p* = 0.05, $n_{p}^{2}=0.075$ ): the Autism group perceived the confederate as more natural and reciprocal than the Typical Group (Figure 3). For naturalness, there was a main effect of Condition, *F*(2,100) = 3.68, *p* = 0.04, $n_{p}^{2}=0.069$ , but there were no effects in the post hoc pairwise comparisons (Figure 3(a)). For reciprocity, there was a main effect of Condition, *F*(2,100) = 37.8, *p* < 0.001, $n_{p}^{2}=0.431$ : the confederate was perceived as more reciprocal in the VideoCall compared to the Video condition, *t*(51) = 5.64, *p* < 0.001, d_z_ = 0.782; more reciprocal in the Real compared to the Video condition, *t*(51) = 6.94, *p* < 0.001, d_z_ = 0.962; and more reciprocal in the Real compared to the VideoCall condition, *t*(51) = 3.93, *p* < 0.001, d_z_ = 0.544 (Figure 3(b)). There was no significant interaction effect between Condition and Group for any of the traits.

### Aggregated analyses

To investigate general patterns of eye gaze and facial motion across the three conditions, we aggregated the data across all time-points and trials for each Condition. Table 3 gives descriptives for these measures.

**Table 3.** Descriptives for aggregated analyses in Autism Study.

Condition	Group	Prop. looking time to Eyes region	Prop. looking time to Mouth region	Number facial AUs
Video	Typical	*M* = 0.127 *SD* = 0.139	*M* = 0.131 *SD* = 0.121	*M* = 4.34 *SD* = 1.37
	Autism	*M* = 0.145 *SD* = 0.126	*M* = 0.101 *SD* = 0.100	*M* = 3.86 *SD* = 0.980
VideoCall	Typical	*M* = 0.086 *SD* = 0.103	*M* = 0.090 *SD* = 0.086	*M* = 4.96 *SD* = 1.51
	Autism	*M* = 0.059 *SD* = 0.066	*M* = 0.061 *SD* = 0.046	*M* = 4.29 *SD* = 1.22
Real	Typical	*M* = 0.064 *SD* = 0.069	*M* = 0.095 *SD* = 0.093	*M* = 4.81 *SD* = 1.59
	Autism	*M* = 0.094 *SD* = 0.113	*M* = 0.074 *SD* = 0.071	*M* = 4.27 *SD* = 1.35

*SD*: standard deviation; AU: action unit.

For eye gaze, we performed a 2-way repeated measures ANOVA with mean proportion of looking time to each ROI (Eyes and Mouth region) as dependent variable, Condition as within-subject factor and Group as between-subject factor. For gaze directed to the Eyes region, there was a main effect of Condition, *F*(2,100) = 9.98, *p* < 0.001, $n_{p}^{2}=0.166$ . Participants looked more to the Eyes region of the confederate in the Video compared to the VideoCall condition, *t*(51) = 3.76, *p* = 0.001, d_z_ = 0.522, and in the Video compared to the Real condition, *t*(51) = 3.35, *p* = 0.006, d_z_ = 0.465, but there were no differences between VideoCall and Real conditions, *t*(51) = 0.583, *p* > 0.05, d_z_ = 0.081 (Figure 3(c)). No other main or interaction effects were significant.

For gaze directed to the Mouth region, there was a main effect of Condition, *F*(2,100) = 3.81, *p* = 0.025, $n_{p}^{2}=0.071$ : participants tended to look less to the Mouth region of the confederate in the VideoCall compared to the Video condition, *t*(51) = 2.41, *p* = 0.052, d_z_ = 0.334, but there were no differences between Video and Real conditions, *t*(51) = 2.13, *p* > 0.05, d_z_ = 0.295, and between VideoCall and Real conditions, *t*(51) = 0.600, *p* > 0.05, d_z_ = 0.083 (Figure 3(d)). No other main or interaction effects were significant.

For facial motion, we fitted a multilevel ANOVA with mean number of facial AUs as dependent variable, Participant as random factor (random intercept), Speech as random factor (random slope), and Condition, Group and Speech as fixed factors. This analysis included 1560 data-points (2 groups, 26 participants/group, 3 conditions/participant, 10 trials/condition). There was a main effect of Condition, *F*(2,1512.0) = 12.54, *p* < 0.001: participants moved their face more in the Real compared to the Video condition, *t*(1516.2) = 3.44, *p* = 0.001, d_z_ = 0.039, but there were no differences between VideoCall compared to the Video condition, *t*(1513.8) = 1.60, *p* > 0.05, d_z_ = 0.018, or between VideoCall and Real condition, *t*(1506.2) = 1.84, *p* > 0.05, d_z_ = 0.021 (Figure 3(e)). No other main or interaction effects were significant.

### Time-course analyses

To study more detailed dynamics of social behaviours, we looked at eye gaze and facial motion patterns along five time-windows in the trial: start of the question/interaction (0–10 s), end of the question (10–22 s), turn-taking (22–24 s), start of the answer (24–32 s) and end of the answer/interaction (32–40 s). For eye gaze, the time-courses were smoothed using a moving average filter of 1 s. For each measure (eye gaze to Eyes and Mouth, and facial motion), we fitted the same ANOVA as in the aggregated analysis and added Time-window as a within-subject factor. The multilevel ANOVA for facial motion now included 7800 data-points (2 groups, 26 participants/group, 3 conditions/participant, 10 trials/condition, 5 time-windows/trial). Although we used the time-windowed data for statistical analyses, the full time-course data are shown on plots. Table 4 gives descriptives for the Typical group, and Table 5 gives descriptives for the Autism group. In the following, we report our main findings; for full results and post hoc tests, see Table S1 (for eye gaze) and Table S2 (for facial motion).

**Table 4.** Descriptives for time-course analyses of Typical group in Autism Study.

Condition	Time-window	Prop. gaze to Eye region	Prop. gaze to Mouth region	Number facial AUs
Video	Start question	*M* = 0.145 *SD* = 0.142	*M* = 0.117 *SD* = 0.134	*M* = 3.39 *SD* = 0.990
	End question	*M* = 0.190 *SD* = 0.216	*M* = 0.208 *SD* = 0.203	*M* = 3.02 *SD* = 0.860
	Turn-taking	*M* = 0.141 *SD* = 0.195	*M* = 0.228 *SD* = 0.237	*M* = 3.12 *SD* = 0.942
	Start answer	*M* = 0.045 *SD* = 0.066	*M* = 0.067 *SD* = 0.088	*M* = 4.67 *SD* = 1.37
	End answer	*M* = 0.077 *SD* = 0.108	*M* = 0.052 *SD* = 0.066	*M* = 4.96 *SD* = 1.62
VideoCall	Start question	*M* = 0.081 *SD* = 0.128	*M* = 0.084 *SD* = 0.094	*M* = 3.80 *SD* = 1.19
	End question	*M* = 0.145 *SD* = 0.178	*M* = 0.153 *SD* = 0.150	*M* = 3.59 *SD* = 1.12
	Turn-taking	*M* = 0.105 *SD* = 0.142	*M* = 0.112 *SD* = 0.150	*M* = 3.73 *SD* = 1.29
	Start answer	*M* = 0.031 *SD* = 0.064	*M* = 0.042 *SD* = 0.088	*M* = 5.23 *SD* = 1.61
	End answer	*M* = 0.045 *SD* = 0.065	*M* = 0.036 *SD* = 0.033	*M* = 5.08 *SD* = 1.64
Real	Start question	*M* = 0.019 *SD* = 0.039	*M* = 0.041 *SD* = 0.065	*M* = 3.73 *SD* = 1.46
	End question	*M* = 0.125 *SD* = 0.132	*M* = 0.199 *SD* = 0.225	*M* = 3.53 *SD* = 1.35
	Turn-taking	*M* = 0.077 *SD* = 0.135	*M* = 0.131 *SD* = 0.161	*M* = 3.98 *SD* = 1.42
	Start answer	*M* = 0.039 *SD* = 0.110	*M* = 0.029 *SD* = 0.037	*M* = 5.07 *SD* = 1.55
	End answer	*M* = 0.041 *SD* = 0.068	*M* = 0.053 *SD* = 0.078	*M* = 5.14 *SD* = 1.82

*SD*: standard deviation; AU: action unit.

**Table 5.** Descriptives for time-course analyses of Autism group in Autism Study.

Condition	Time-window	Prop. gaze to Eye region	Prop. gaze to Mouth region	Number facial AUs
Video	Start question	*M* = 0.138 *SD* = 0.112	*M* = 0.107 *SD* = 0.109	*M* = 3.77 *SD* = 1.40
	End question	*M* = 0.223 *SD* = 0.197	*M* = 0.159 *SD* = 0.176	*M* = 3.41 *SD* = 1.44
	Turn-taking	*M* = 0.222 *SD* = 0.209	*M* = 0.160 *SD* = 0.177	*M* = 3.62 *SD* = 1.61
	Start answer	*M* = 0.066 *SD* = 0.086	*M* = 0.044 *SD* = 0.055	*M* = 5.29 *SD* = 1.62
	End answer	*M* = 0.077 *SD* = 0.090	*M* = 0.040 *SD* = 0.045	*M* = 5.56 *SD* = 1.56
VideoCall	Start question	*M* = 0.064 *SD* = 0.066	*M* = 0.066 *SD* = 0.059	*M* = 4.39 *SD* = 1.63
	End question	*M* = 0.087 *SD* = 0.095	*M* = 0.096 *SD* = 0.085	*M* = 4.08 *SD* = 1.66
	Turn-taking	*M* = 0.065 *SD* = 0.079	*M* = 0.076 *SD* = 0.094	*M* = 4.66 *SD* = 1.63
	Start answer	*M* = 0.030 *SD* = 0.065	*M* = 0.027 *SD* = 0.036	*M* = 5.88 *SD* = 1.62
	End answer	*M* = 0.034 *SD* = 0.068	*M* = 0.030 *SD* = 0.053	*M* = 6.04 *SD* = 1.59
Real	Start question	*M* = 0.054 *SD* = 0.061	*M* = 0.035 *SD* = 0.037	*M* = 4.30 *SD* = 1.72
	End question	*M* = 0.185 *SD* = 0.193	*M* = 0.150 *SD* = 0.157	*M* = 3.75 *SD* = 1.67
	Turn-taking	*M* = 0.138 *SD* = 0.0195	*M* = 0.133 *SD* = 0.164	*M* = 4.63 *SD* = 1.76
	Start answer	*M* = 0.037 *SD* = 0.082	*M* = 0.027 *SD* = 0.036	*M* = 5.76 *SD* = 1.86
	End answer	*M* = 0.048 *SD* = 0.092	*M* = 0.033 *SD* = 0.041	*M* = 5.91 *SD* = 1.85

*SD*: standard deviation; AU: action unit.

**Figure 4.**
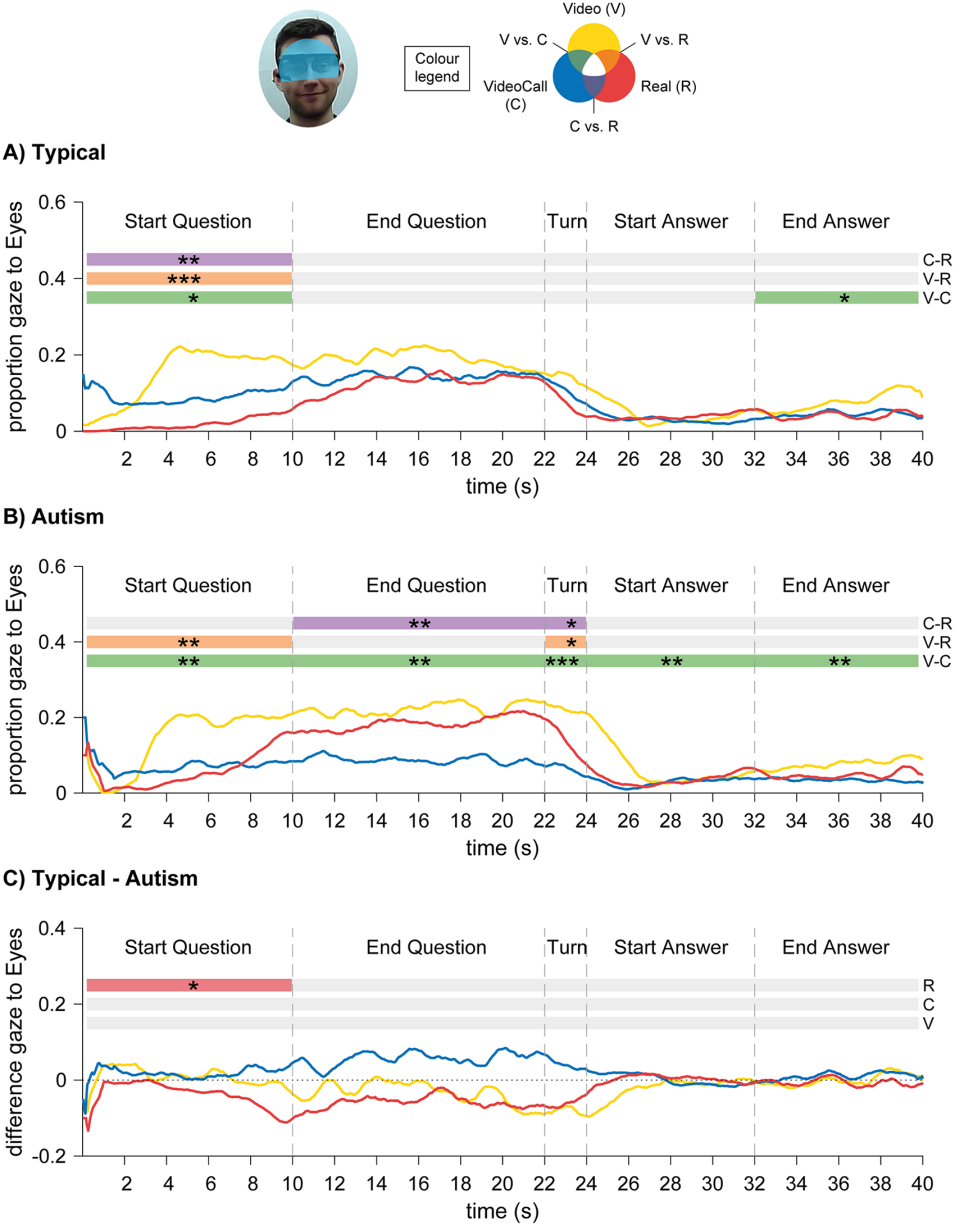
Results for time-course analyses of eye gaze directed to Eyes region in Autism Study: (a) Typical group; (b) Autism group. (c) Difference between Typical and Autism groups: positive values indicate Typical > Autism, and negative values indicate Autism > Typical. Asterisks signify difference at *p* < 0.05 (*), *p* < 0.01 (**) and *p* < 0.001 (***).

For gaze directed to the Eyes region of the confederate, there was a main effect of Condition, *F*(2,100) = 10.4, *p* < 0.001, $n_{p}^{2}=0.172$ , and a main effect of Time-window, *F*(4,200) = 39.7, *p* < 0.001, $n_{p}^{2}=0.443$ : participants looked more to the eyes of the confederate in the Video than in the VideoCall and Real conditions, and during the Question phases than during Turn-taking and Answer phases. There was an interaction effect between Condition and Time-window, *F*(8,400) = 5.55, *p* < 0.001, $n_{p}^{2}=0.100$ , and an interaction between Condition, Time-window and Group, *F*(8,400) = 2.81, *p* = 0.028, $n_{p}^{2}=0.053$ . At the start of the Question phase, both groups looked less to the Eyes region in the VideoCall and Real conditions (compared to the Video), and at the end of the Answer phase they looked less in the VideoCall condition (see Figure 4(a) and (b)). Between-group differences in the Real condition revealed that, at the start of the Question phase, the Typical group looked less to the Eyes region than the Autism group (see Figure 4(c)). No other main or interaction effects were significant.

**Figure 5.**
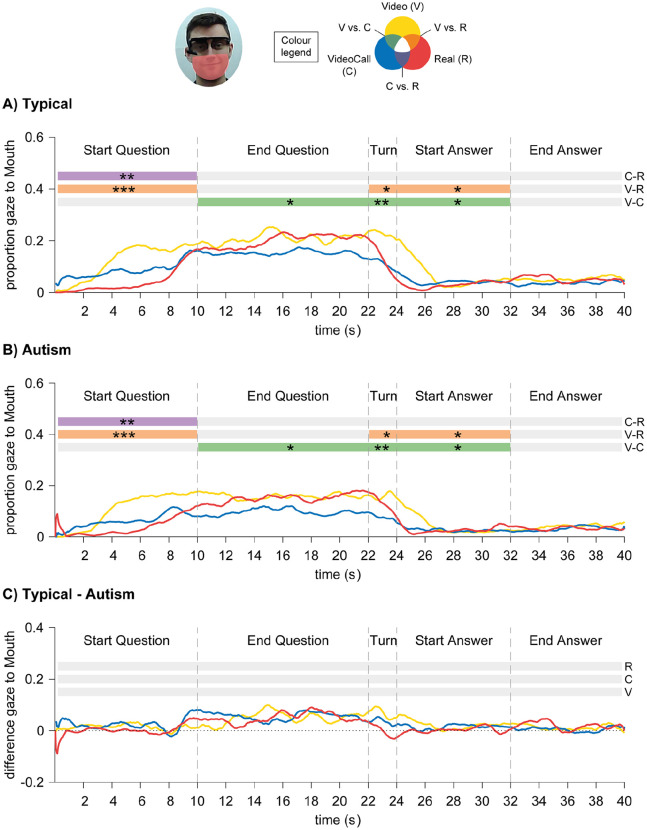
Results for time-course analyses of eye gaze directed to Mouth region in Autism Study: (a) Typical group; (b) Autism group. (c) Difference between Typical and Autism groups: positive values indicate Typical > Autism, and negative values indicate Autism > Typical. Asterisks signify difference at *p* < 0.05 (*), *p* < 0.01 (**) and *p* < 0.001 (***).

For gaze directed to the Mouth region of the confederate, there was a main effect of Condition, *F*(2,100) = 4.83, *p* = 0.01, $n_{p}^{2}=0.088$ , and a main effect of Time-window, *F*(4,200) = 38.7, *p* < 0.001, $n_{p}^{2}=0.437$ : participants looked more to the mouth of the confederate in the Video than in the VideoCall condition, and during the Question phase than during Turn-taking and Answer phase. There was an interaction effect between Condition and Time-window, *F*(8,400) = 4.86, *p* = 0.002, $n_{p}^{2}=0.089$ . For both groups, at the start of the Question phase participants looked less to the Mouth region in the Real condition (compared to VideoCall and Video), and looked less at Turn-taking and start of Answer phase in the VideoCall and Real conditions (compared to Video) (see Figure 5(a) and (b)). No other main or interaction effects were significant.

**Figure 6.**
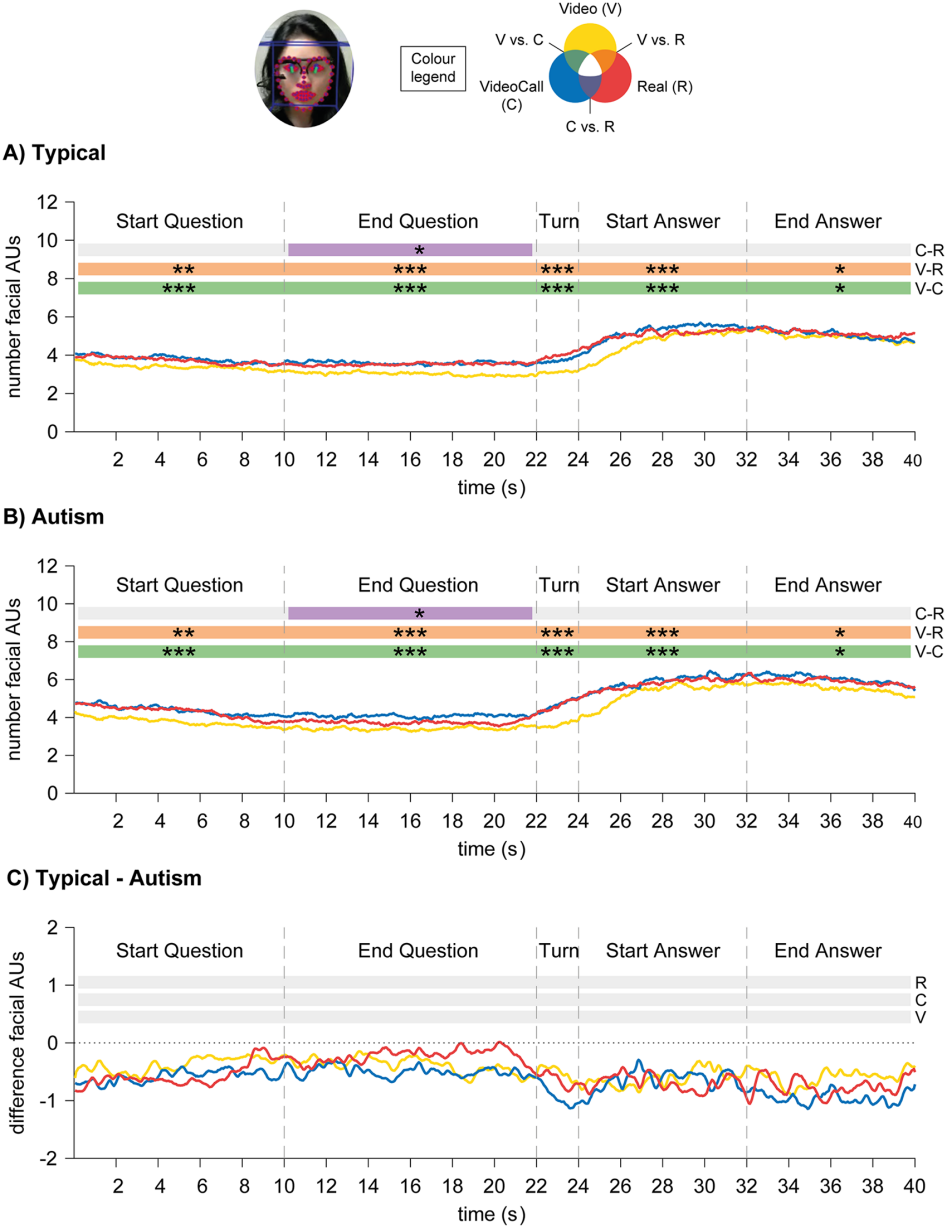
Results for time-course analyses of facial motion in Autism Study: (a) Typical group; (b) Autism group. (c) Difference between Typical and Autism groups: positive values indicate Typical > Autism, and negative values indicate Autism > Typical. Asterisks signify difference at *p* < 0.05 (*), *p* < 0.01 (**) and *p* < 0.001 (***).

For facial motion, there was a main effect of Condition, *F*(2,7657.5) = 59.0, *p* < 0.001, and a main effect of Time-window, *F*(4,7669.2) = 76.0, *p* < 0.001: participants moved their face more in the Real condition than in the Video condition, and during the Answer phase than during the Question phase and Turn-taking (see Figure 6(a) and (b)). There was also an interaction effect between Condition and Time-window, *F*(8,7653.5) = 1.99, *p* = 0.043, but there were no effects in the post hoc pairwise comparisons. No other main or interaction effects were significant.

## Discussion

We investigated how typical and autistic gaze patterns are modulated by the belief in being watched and potential to show true gaze direction during a Q&A task. We also performed an exploratory analysis to test these effects on facial motion patterns. Contrary to what we expected, typical and autistic participants showed similar modulation of eye gaze and facial displays: they looked less to the confederate and produced more facial displays when being watched and when speaking. However, at the start of the face-to-face interaction, autistic participants gazed more to the confederate’s eyes than typical participants. These findings challenge previous studies reporting atypical gaze behaviour in autism.

### Social signalling in typical individuals

To investigate general patterns of eye gaze, we aggregated the data across the time-courses for each condition. Replicating the Pilot Study, participants looked less to the eyes of the confederate in the Real and VideoCall conditions compared to the Video condition. These findings are consistent with previous studies showing that participants gaze less to a live partner than to a video-clip of the same partner, either if they are actively interacting (Cañigueral & Hamilton, 2019a) or not (Laidlaw et al., 2011). The similar pattern of gaze-to-eyes across VideoCall and Real conditions also suggests that participants were able to resolve the mismatch between true and perceived gaze direction in the VideoCall condition. We also found that participants tended to look less to the mouth of the partner in the VideoCall than in the Video condition, although this effect did not reach significance.

To fully understand which cognitive mechanisms modulate gaze planning in live interactions, it is necessary to examine how eye gaze changes along the interaction in relation to other signals, such as speech. Consistent with the Pilot Study, we found that participants looked more to the eyes and mouth of the confederate during the Question phase than during the Answer phase. This is in line with previous studies investigating the regulatory function of gaze (Hessels et al., 2019; Ho et al., 2015; Kendon, 1967), which found that participants look more to the partner when listening than when speaking. However, this modulation was also true for the Video condition, where participants knew the confederate was pre-recorded. This could indicate that we avert gaze while speaking to reduce cognitive demands linked to looking at faces (Beattie, 1981; Glenberg et al., 1998; Kendon, 1967; Markson & Paterson, 2009). Future studies will be needed to clarify this finding.

Similar to the Pilot Study, time-course analyses showed that gaze-to-eyes and gaze-to-mouth patterns in the three conditions differed along the trial time-course. At the start of the Question phase, participants looked less to the eyes and mouth of the confederate in the live conditions (VideoCall and Real). The fact that participants especially averted gaze-to-eyes when there could be true direct gaze (Real condition) suggests that during live interactions participants averted gaze to reduce arousal associated with making eye contact (Kendon, 1967; Pönkänen et al., 2011). Another possibility for more gaze-to-mouth in the Video condition is that participants relied more strongly on lip-reading to make sure they fully understood the question, since they only had one chance to hear it (in the VideoCall and Real conditions participants could have asked for brief clarifications, and the confederate reported that this happened a few times). At turn-taking and start of the Answer phase, participants gazed less to the confederate’s mouth in the VideoCall and Real conditions, suggesting that participants disengaged faster from the mouth of the live confederate than the pre-recorded confederate. Again, this could either be because gaze has a regulatory function (e.g. avert gaze when starting to speak) or because live faces are more cognitively demanding than pre-recorded faces. Studying the role of cognitive load associated with live faces and live interactions can shed some light on this question. Moreover, the similar gaze patterns found across the live conditions indicate that participants could adjust to the discrepancy between true and perceived gaze direction in the VideoCall condition.

To complement our gaze findings, we also looked at patterns of facial motion across the three conditions while controlling for effects related to speech production. As in the Pilot Study, participants moved their face more in the Real and VideoCall conditions than in the Video condition for the whole time-course, and this effect was particularly marked during turn-taking. Participants also moved their face more during the Answer phase than during the Question phase. In line with previous studies, this indicates that participants used facial displays as social signals (Chovil, 1991b; Crivelli & Fridlund, 2018; Fridlund, 1991; Hietanen et al., 2019).

### Social signalling in autistic individuals

Contrary to what we expected, general patterns of gaze-to-eyes and gaze-to-mouth in the aggregated analysis were the same between typical and autistic groups. To our knowledge, this is the first study to systematically compare gaze patterns of clinically diagnosed autistic adults in live versus pre-recorded interactions, and our findings suggest that gaze planning in autism is modulated by both its perceiving and signalling functions. In contrast with these findings, a previous study (Von dem Hagen & Bright, 2017 Experiment 1) found that typical participants with high autistic traits directed equal gaze to a live video-feed and a pre-recorded video. A key difference is that in their study participants were not clinically diagnosed as autistic. It could be that clinically diagnosed individuals can better understand their difficulties and improve the management of their social behaviour. This may help them to develop compensation strategies, that is, show improved behavioural presentation of symptoms although deficits persist at the cognitive and neurobiological level (Livingston & Happé, 2017). Another difference is that in Von dem Hagen and Bright’s study participants were not actively engaged with the person in the video-feed: being in an explicit communicative context, such as the one in the present study, may be a cue for autistic participants to use eye gaze as a social signal.

For the time-course analysis of gaze, we found that typical and autistic participants showed overall similar patterns of gaze-to-eyes and gaze-to-mouth. Consistent with previous studies (Freeth & Bugembe, 2019; Vabalas & Freeth, 2016; Von dem Hagen & Bright, 2017 Experiment 2), both groups gazed more to the confederate when listening than when speaking. This suggests that high-functioning autistic individuals are able to modulate gaze behaviour according to their role in the conversation (speaker or listener). Moreover, at the start of the interaction and at turn-taking autistic participants looked less to the eyes and mouth of the confederate when they were being watched (VideoCall or Real condition) than when not being watched (Video condition). This indicates that in live interactions autistic participants may also avert gaze to reduce arousal (Argyle & Dean, 1965; Pönkänen et al., 2011), to regulate the interaction (Kendon, 1967) or to reduce cognitive demands of looking at faces (Beattie, 1981; Glenberg et al., 1998; Markson & Paterson, 2009). However, we cannot distinguish whether this reflects spontaneous gaze behaviour or compensation strategies (Livingston & Happé, 2017).

Direct comparison between typical and autistic gaze patterns revealed that, only in the Real condition and at the start of the Question phase, autistic participants directed *more* gaze to the eyes of the confederate than the typical group. This evidence challenges previous studies showing that autistic participants use eye gaze similarly to typical individuals during a live Q&A task (Freeth & Bugembe, 2019; Vabalas & Freeth, 2016; Von dem Hagen & Bright, 2017 Experiment 2), or that they spend less time looking at a live confederate (Hessels et al., 2018; Von dem Hagen & Bright, 2017 Experiment 2). Interestingly, two recent studies have found that participants with high social anxiety traits look earlier and more to faces at the start of the interaction, compared to participants with low social anxiety traits (Gregory et al., 2019; Gutiérrez-García et al., 2019). The authors suggest that this attentional bias could reflect compensation strategies to anticipate negative evaluations. In our study, the initial attentional bias could also reflect a compensation strategy, where autistic participants have learnt that they need to make more eye contact during face-to-face interactions (Del Bianco et al., 2018; Livingston & Happé, 2017).

Overall, our findings suggest that autistic individuals do *not* have reduced interest to attend to other people’s faces, and that they are generally able to adjust eye gaze to the demands of a structured conversation. This contradicts the social motivation theory (Chevallier et al., 2012) and the dialectal misattunement hypothesis (Bolis et al., 2018) of autism. However, the possibility that gaze patterns are slightly different in spontaneous conversations and that autistic participants are using compensation strategies makes it hard to draw conclusive interpretations from our findings. Further research will be needed to clarify *if* and *how* gaze patterns in spontaneous face-to-face interactions support each of these theories.

Finally, we examined facial motion patterns in autistic individuals. Contrary to previous studies (Trevisan et al., 2018), both aggregated and time-course analyses yielded no differences between typical and autistic groups: autistic participants also showed more facial motion when being watched and when speaking, suggesting that they use facial displays as a social signal (Chovil, 1991a; Crivelli & Fridlund, 2018). A limitation to this finding is that we do not have information about the content of facial displays: studying whether facial displays are meaningful or not to the spoken message will be an interesting question for future research.

### Limitations and future research

A main limitation in our study is that the task we used was very structured and missed the continuity of natural conversations. Although both typical and autistic participants rated the confederate as increasingly reciprocal across Video, VideoCall and Real conditions, there were no differences in ratings of naturalness. Moreover, some of our findings (e.g. averting gaze at start and end of live interactions, no overall differences between typical and autistic groups) could be explained by the structured nature of our task. Similarly, our experimental set-up used an occluder, which could limit the ecological validity of our results. Using a task where confederate and participants engage in natural conversation in a more ecologically valid set-up could provide further insight into how eye gaze is used in real life. Nonetheless, the fact that there are no major differences in gaze patterns between VideoCall and Real conditions validates the use of video-calls as a reliable setting to simulate face-to-face interactions in research studies.

Another limitation is that we could not use the eye-tracking data from the confederate, since recordings had poor signal quality, so we could not check whether gaze patterns of the confederate were the same for the typical and autistic group. This also restricts investigations about how patterns of eye gaze are related between interacting partners, or how much eye contact confederate and participant are making: this could provide further insight about how they use social signals to communicate with each other. Furthermore, our sample only included high-functioning autistic individuals: it will be important to test to what extent our findings hold across the spectrum.

The present study opens up several questions for future research. For instance, an interesting question is how gaze patterns are related to cognitive demands associated to looking at (live) faces (Beattie, 1981; Glenberg et al., 1998; Markson & Paterson, 2009) or the conversation topic (Hutchins & Brien, 2016; Nadig et al., 2010). Including reliable measures of response latency (to assess the difficulty of the task) and executive functions could contribute to clarify this question. Another question for future research is to what extent autistic individuals use compensation strategies to guide gaze behaviour during social interactions (Livingston & Happé, 2017). Here, we have shown how using time-course analysis is helpful to pinpoint specific differences between typical and autistic groups (e.g. at the start of the interaction). Designing more elaborate paradigms in ecologically valid environments and using more fine-grained analyses could help identify which cognitive components of gaze processing are disrupted in autism. Finally, our findings show that, although participants displayed similar gaze patterns in the VideoCall and Real conditions, they perceived the VideoCall condition as less reciprocal than the Real condition. This raises the question of what gives a feeling of reciprocity in face-to-face interactions. We suggest that delays in the video-call connection probably hinder subtle but fundamental aspects of face-to-face interactions, for example, interpersonal coordination of body movements, although future studies will be needed to clarify this question.

## Conclusion

The present study investigated how gaze behaviour in typical and autistic individuals is modulated by the belief in being watched and potential to show true gaze direction. Contrary to our hypotheses, gaze patterns were overall similar across typical and autistic groups: both groups gazed less to the confederate when being watched and when speaking. However, at the start of a face-to-face interaction, autistic participants gazed more to the confederate’s eyes than typical participants. An exploratory analysis also suggested that both groups used facial displays as a social signal. These findings indicate that the use of social signals in autism is less compromised than previously reported.

## Supplemental Material

draft_v9_Autism_supplmat – Supplemental material for Effects of being watched on eye gaze and facial displays of typical and autistic individuals during conversation Supplemental material, draft_v9_Autism_supplmat for Effects of being watched on eye gaze and facial displays of typical and autistic individuals during conversation by Roser Cañigueral, Jamie A Ward and Antonia F de C Hamilton in Autism
