# Supplementary material for: Effects of being watched on eye gaze and facial displays of typical and autistic individuals during conversation
Source: Autism. 2020 Aug 27;25(1):210–26. doi: 10.1177/1362361320951691 (PMC7812513; doi:10.1177/1362361320951691)
Supplement: draft_v9_Autism_supplmat – Supplemental material for Effects of being watched on eye gaze and facial displays of typical and autistic individuals during conversation [file draft_v9_Autism_supplmat.pdf]

Supplementary Materials

S1. Pilot Study

Hypotheses

The Pilot Study investigated eye gaze and facial motion patterns of typical participants while they completed a Q&A task in three social contexts: Video, VideoCall and Real. Based on previous studies (Cañigueral & Hamilton, 2019a; Laidlaw et al., 2011), for the aggregated analysis of eye gaze we expected that participants would direct less gaze towards the confederate in the VideoCall and Real conditions (where gaze has a perceiving and signalling function) compared to the Video condition (where gaze can only perceive information). We predicted no differences between VideoCall and Real conditions: these two conditions differ on the potential to show true gaze direction, but effects of this subtle manipulation are probably hard to capture using aggregated measures.

For the time-course analysis of eye gaze, we looked at differences between conditions in 5 different time-windows along the trial time-course: start of question, end of question, turn-taking, start of answer, and end of answer. In line with previous evidence (Ho et al., 2015; Kendon, 1967), we predicted that participants would direct more gaze to the confederate during the question time-windows (i.e. when they were listening) than during the answer time-windows (i.e. when they were speaking). We expected this pattern would be true particularly for the VideoCall and Real conditions, where gaze acquires a signalling function and eye movements are planned to regulate turn-taking. Moreover, we expected that differences between Real and VideoCall (or Video) conditions would be greater at moments where gaze is planned, not only to monitor information about the other, but also to signal information about who is taking the turn. Thus, based on previous studies, we predicted that participants would gaze more to the confederate in the Real condition at the

start of the Question (to signal interest in what the confederate was saying; Argyle & Cook, 1976; Kendon, 1967), during Turn-taking (to signal that they are taking the turn; Ho et al., 2015; Kendon, 1967), and at the end of the Answer (to signal that they were ending the answer and to monitor what the confederate thinks about their answer; Efran, 1968; Efran & Broughton, 1966; Ho et al., 2015; Kendon, 1967; Kleinke, 1986).

Finally, if participants use facial displays as a tool for communication (Crivelli & Fridlund, 2018), we predicted they would generally move their face more in the VideoCall and Real conditions compared to the Video condition. Moreover, since we use facial displays to add meaning to speech (Chovil, 1991a), we expected that along the trial time-course participants would move their face more during the answer time-windows (i.e. when they were speaking) than during the question time-windows (i.e. when they were listening). A potential confound in this analysis is that the face-tracking algorithm may pick up facial motion related to moving the mouth when speaking: this limitation was addressed in the Autism Study.

Materials and Methods

Participants and confederate

Thirty healthy adult participants were recruited using a participant database at the author's institution and participated in the study (25 females, 5 males; mean age: 22.93±2.78; specific data on ethnicity, socioeconomic status and educational attainment levels were not recorded). This sample size was chosen based on previous studies using similar Q&A tasks (e.g. Cañigueral & Hamilton, 2019a; Freeth, Foulsham, & Kingstone, 2013), and was later used to run a power analysis and specify the sample size in the Autism Study. Two participants were excluded from the analyses due to poor signal quality in the eye-tracking data, so the final sample consisted of a group of 28 adults (23 females, 5 males;

mean age: 22.96±2.87). The confederate was a professional actress (playing age: 18-28) hired for the full duration of the study, to ensure a consistent performance between trials and participants. Importantly, she was unaware of the aims and hypotheses of the study. Participants were told the confederate was a student helping with the study. All participants and the confederate provided written informed consent and were compensated for their participation in the study. The study was granted ethical approval by the local Research Ethics Committee, and was in accordance with the Declaration of Helsinki.

Task

Participants completed the same task as in the Autism Study (see Figure 1B). However, the 3 sets of questions comprised 12 items (instead of 10). See Supplementary Materials (S2) for the full list of questions used in the Pilot Study.

Experimental conditions and stimuli

Participants completed the task under the same three conditions as in the Autism Study. All videos for the Video condition were recorded with the confederate for the Pilot Study, and the behaviour of the confederate during the task was the same one as in the Autism Study. Note that we counterbalanced the order of the experimental conditions, creating 6 different counterbalancing conditions: *V-C-R, V-R-C, C-V-R, C-R-V, R-V-C, R-C-V.* Each participant was allocated to one counterbalancing condition and completed the task under each of the three experimental conditions. The overall duration of the study was around 45 minutes.

Post-test questionnaire and debriefing

After completing the task under the three conditions, all participants completed the same post-test questionnaire as in the Autism Study (see Supplementary Materials S3 for the full post-test questionnaire), and were debriefed about the real purpose of the study.

Experimental set-up

The experimental set-up was the same one as in the Autism Study, except that in the Pilot Study we did not use lapel microphones (see Figure 1A).

Eye gaze and facial motion data: acquisition and processing

The acquisition and processing of eye gaze and facial motion data was the same as in the Autism Study. Note that no speech data was recorded nor processed in the Pilot Study.

Statistical analyses

To check whether our experimental manipulation modulated how participants perceived the interaction with the confederate, a 1-way repeated measures ANOVA with Condition (Video, VideoCall, Real) as within-subject factor was performed for each of the traits rated in the post-test questionnaire: naturalness and reciprocity. Where sphericity could not be assumed, corrected p-values using the Greenhouse-Geisser estimate were used. Post-hoc pairwise comparisons using Bonferroni's adjustment were also computed.

For eye gaze and facial motion data, we performed two types of analyses: aggregated and time-course. Aggregated analyses are useful to investigate general patterns of behaviour across different conditions, and use aggregated data across entire recording sessions (see Figure 2). For the eye gaze analysis, we computed the mean proportion of looking time for each ROI (Eyes and Mouth) and Condition, across all time-points and trials. Note that proportion of looking time refers to the amount of time that participants spent looking at each ROI, relative to the total duration of the trial. For the facial motion analysis, we computed the mean number of active facial AUs for each Condition, across all time-points and trials. As before, for each measure (proportion looking time to Eyes, proportion looking time to Mouth, and number facial AUs), we performed a 1-way repeated measures ANOVA with Condition (Video, VideoCall, Real) as within-subject factor.

Although aggregated analyses yield important insight into patterns of behaviour, time-course analyses offer the possibility to study more fine-grained dynamics of behaviour along time, which are otherwise lost in aggregated analyses (see Figure 2). Here, our aim was to investigate how eye gaze and facial motion patterns vary throughout the time-course of the trial in relation to speech. For this, we distinguished between 5 different time-windows in the trial: start of the question/interaction (0-10 s), end of the question (10-20 s), turn-taking (20-24 s), start of the answer (24-32 s), and end of the answer/interaction (32-40 s). Note that the time-window for turn-taking was 4 seconds, which is a rather long duration for this type of event. We chose this longer time-window because, since we did not have an accurate time measurement for the end of each question in the VideoCall and Real conditions (which slightly varied across participants), we used the time values from a pre-recorded version of these questions (with the same confederate). This means that, although the time-course for each trial was locked to the end of the Question phase according to these time values (i.e. around 22 s), for some trials this event might have happened slightly earlier or later than the values used. Thus, we chose a time-window at 22±2 s to account for this variability.

For the eye gaze analysis, we computed the mean proportion of gaze for each ROI and time-point, across trials in the same Condition. Thus, we obtained two time-courses (gaze to Eyes and gaze to Mouth) for each participant and Condition, with the mean proportion of gaze to each ROI along the trial duration. These time-courses were smoothed using a moving average filter of 1 second. For the facial motion analysis, we computed the mean number of facial AUs for each time-point, across trials in the same Condition: we obtained one time-course for each participant and Condition, with the mean number of facial AUs along the trial duration. For each measure (proportion gaze to Eyes, proportion

gaze to Mouth, and number facial AUs), we computed the means for each time-window. A 2-way repeated measures ANOVA with Condition (Video, VideoCall, Real) and Time-window (Start Question, End Question, Turn-taking, Start Answer and End Answer) as within-subject factors was performed for each measure. Note that, although we used the time-window data for statistical analyses, the full time-course data was used for plots.

Results

Manipulation check: post-test questionnaire ratings

In the post-test questionnaire, participants rated the confederate in each condition on two traits: naturalness and reciprocity. A 1-way repeated measures ANOVA with Condition (Video, VideoCall, Real) as within-subject factor was performed for each of the traits. See Table S1-1 for descriptives (mean and SD) on post-test questionnaire ratings. For naturalness, results showed that there was a main effect of Condition, $F(2,54) = 5.037$, $p < .05$, $n_p^2 = .157$: the confederate was perceived as more natural in the VideoCall compared to the Video condition, $t(27) = 3.02$, $p < .05$, $d_z = .570$, but there was no difference between Video and Real conditions, $t(27) = 2.37$, $p > .05$, $d_z = .449$, and between VideoCall and Real conditions, $t(27) = .449$, $p > .05$, $d_z = .084$ (Figure S1-1A). For reciprocity, results showed that there was a main effect of Condition, $F(2,54) = 14.2$, $p < .001$, $n_p^2 = .345$: the confederate was perceived as more reciprocal in the VideoCall compared to the Video condition, $t(27) = 3.10$, $p < .05$, $d_z = .586$; more reciprocal in the Real compared to the Video condition, $t(27) = 4.16$, $p < .001$, $d_z = .787$; and more reciprocal in the Real compared to the VideoCall condition, $t(27) = 3.52$, $p < .01$, $d_z = .665$ (Figure S1-1A).

Table S1-1. Descriptives for post-test questionnaire ratings in Pilot Study. Scale 0 (disagree) to 8 (agree).

Condition	Natural	Reciprocal
Video	*M* = 5.04<br>*SD* = 2.01	*M* = 4.04<br>*SD* = 2.43
VideoCall	*M* = 5.71<br>*SD* = 1.80	*M* = 4.89<br>*SD* = 2.35
Real	*M* = 5.82<br>*SD* = 2.07	*M* = 5.57<br>*SD* = 2.47

Aggregated analyses

To investigate general patterns of eye gaze and facial motion across the three conditions, we aggregated the data across all time-points and trials for each Condition. Using the proportion of looking time to Eyes and Mouth region as measures for eye gaze, and the number of facial AUs as a measure for facial motion, we fitted a 1-way repeated measures ANOVA with Condition (Video, VideoCall, Real) as within-subject factor. See Table S1-2 for descriptives (mean and SD) on these measures.

For eye gaze directed to the Eyes region, there was a main effect of Condition, $F(2,54) = 7.003$, $p < .01$, $n_p^2 = .206$. Post-hoc pairwise comparisons showed that participants looked less to the Eyes region of the confederate in the Real compared to the Video condition, $t(27) = 3.62$, $p < .01$, $d_z = .684$, but there was no difference between Video and VideoCall conditions, $t(27) = 2.52$, $p > .05$, $d_z = .477$, and between VideoCall and Real conditions, $t(27) = 1.15$, $p > .05$, $d_z = .217$ (Figure S1-1B). However, since we detected an outlier in the VideoCall condition, we repeated the analysis after removal of this outlier for all conditions. Results showed that there was a main effect of Condition, $F(2,52) = 9.113$, $p < .01$, $n_p^2 = .260$. Post-hoc pairwise comparisons showed that participants looked less to the Eyes region of the confederate in the Real compared to the Video condition, $t(26) = 3.45$, $p < .01$, $d_z = .664$, and in the VideoCall compared to the Video condition, $t(26) = 3.15$, $p < .05$, d_z

= .606 (see blue asterisk in Figure S1-1B). There was no difference between VideoCall and Real conditions, $t(26) = .583$, $p > .05$, $d_z = .112$.

Table S1-2. Descriptives for aggregated analyses in Pilot Study

**Condition**	**Prop. looking time to Eyes region**[a]	**Prop. looking time to Mouth region**	**Number facial AUs**
Video	*M* = .123 (.116)<br>*SD* = .109 (.105)	*M* = .160<br>*SD* = .096	*M* = 4.16<br>*SD* = 1.09
VideoCall	*M* = .070 (.053)<br>*SD* = .107 (.061)	*M* = .095<br>*SD* = .065	*M* = 4.43<br>*SD* = 1.32
Real	*M* = .047 (.046)<br>*SD* = .049 (.050)	*M* = .118<br>*SD* = .093	*M* = 4.62<br>*SD* = 1.56

[a]Values after removal of the outlier are in brackets.

For eye gaze directed to the Mouth region, there was a main effect of Condition, $F(2,54) = 4.01$, $p < .05$, $n_p^2 = .129$: participants looked less to the Mouth region of the confederate in the VideoCall compared to the Video condition, $t(27) = 3.09$, $p < .05$, $d_z = .585$, but there was no difference between Video and Real conditions, $t(27) = 1.5$, $p > .05$, $d_z = .294$, and between VideoCall and Real conditions, $t(27) = 1.04$, $p > .05$, $d_z = .197$ (Figure S1-1B).

For facial motion, there was a main effect of Condition, $F(2,54) = 4.33$, $p < .05$, $n_p^2 = .138$: participants moved their face more in the Real compared to the Video condition, $t(27) = 2.91$, $p < .05$, $d_z = .551$, but there was no difference between Video and VideoCall conditions, $t(27) = 2.06$, $p > .05$, $d_z = .389$, and between VideoCall and Real conditions, $t(27) = 1.03$, $p > .05$, $d_z = .194$ (Figure S1-1C).

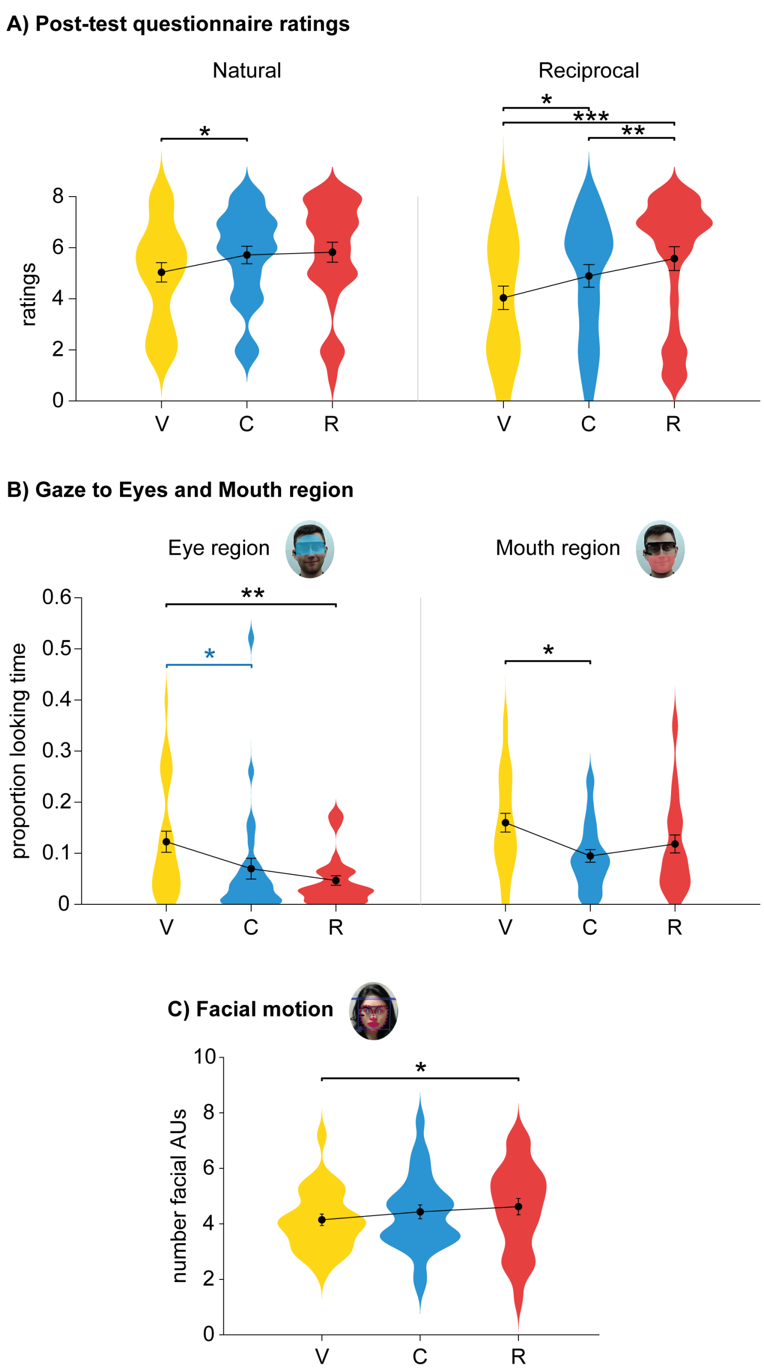

Figure S1-1. Results for ratings and aggregated analyses of eye gaze in Pilot Study. A) Post-test questionnaire ratings. B) Proportion of looking time to Eyes and Mouth region for each Condition. C) Number of facial AUs for each Condition. Mean (filled circle), SE (error bars), and frequency of values (width of distribution). Asterisks signify difference at $p < .05$ (*), $p < .01$ (**) and $p < .001$ (***). Blue asterisks signify difference between V-C after removing the outlier. V = Video, C = VideoCall, R = Real.

Time-course analyses

Using time-course analyses, we aimed to study more detailed dynamics of eye gaze and facial motion along the trial, which cannot be captured by aggregated analyses. For each measure (proportion gaze to Eyes, proportion gaze to Mouth, number of facial AUs), we computed the mean along the time-course, across trials in the same Condition. We distinguished between 5 different time-windows in the trial, and performed a 2-way repeated measures ANOVA with Condition (Video, VideoCall, Real) and Time-window (Start Question, End Question, Turn-taking, Start Answer and End Answer) as within-subject factors. Only significant main effects and interactions are reported in the text; see Table S1-3 for descriptives (mean and SD), and Table S1-4 for full results and post-hoc tests.

For eye gaze directed to the Eyes region of the confederate, there was a main effect of Condition, $F(2,54) = 6.82$, $p < .01$, $n_p^2 = .202$, and a main effect of Time-window, $F(4,108) = 21.9$, $p < .001$, $n_p^2 = .447$: participants generally looked more to the eyes of the confederate in the Video than in the Real condition (after removing the outlier, only more gaze in the Video than in the VideoCall condition), and during the Question phase than during Turn-taking and Answer phase. There was also an interaction effect between Condition and Time-window, $F(8,216) = 4.81$, $p < .01$, $n_p^2 = .151$. At the start of the Question phase participants looked less to the Eyes region in the VideoCall and Real conditions (compared to the Video), and a similar pattern was found at the end of the Question phase and at Turn-taking for the Real condition (but only for the VideoCall condition after removing the outlier). At the end of the Answer phase participants increased gaze directed to the eyes in the Video condition (compared to VideoCall and Real), as well as in the VideoCall condition compared to the Real condition (see Figure S1-2A).

For eye gaze directed to the Mouth region of the confederate, there was a main effect of Condition, $F(2,54) = 4.17$, $p < .05$, $n_p^2 = .134$, and a main effect of Time-window, $F(4,108) = 44.2$, $p < .001$, $n_p^2 = .621$: participants generally looked more to the mouth of the confederate in the Video than in the VideoCall condition, and during the Question phase than during Turn-taking and Answer phase. There was also an interaction effect between Condition and Time-window, $F(8,216) = 3.52$, $p < .05$, $n_p^2 = .115$. At the start of the Question phase participants looked less to the Mouth region in the VideoCall and Real conditions (compared to the Video), and a similar pattern was found at the end of the Question phase and at Turn-taking for the VideoCall condition. At the end of the Answer phase participants increased gaze directed to the eyes in the Video condition (compared to VideoCall and Real) (see Figure S1-2B).

For facial motion, there was a main effect of Condition, $F(2,54) = 4.63$, $p < .05$, $n_p^2 = .146$, and a main effect of Time-window, $F(4,108) = 110.2$, $p < .001$, $n_p^2 = .803$: participants generally moved their face more in the Real than in the Video condition, and during the Answer phase than during the Question phase and Turn-taking. There was no interaction effect between Condition and Time-window, $F(8,216) = 2.60$, $p > .05$, $n_p^2 = .088$ (Figure S1-2C).

Table S1-3. Descriptives for time-course analyses in Pilot Study

Condition	Time-window	Prop. gaze to Eye region[a]	Prop. gaze to Mouth region	Number facial AUs
Video	Start Question	*M* = .189 (.182)<br>*SD* = .148 (.141)	*M* = .221<br>*SD* = .140	*M* = 3.39<br>*SD* = 1.13
	End Question	*M* = .160 (.152)<br>*SD* = .150 (.150)	*M* = .258<br>*SD* = .177	*M* = 3.12<br>*SD* = 1.12
	Turn-taking	*M* = .075 (.072)<br>*SD* = .121 (.121)	*M* = .151<br>*SD* = .126	*M* = 3.42<br>*SD* = 1.18
	Start Answer	*M* = .037 (.031)<br>*SD* = .055 (.040)	*M* = .031<br>*SD* = .038	*M* = 5.78<br>*SD* = 1.27
	End Answer	*M* = .103 (.093)<br>*SD* = .132 (.116)	*M* = .090<br>*SD* = .085	*M* = 5.13<br>*SD* = 1.38
VideoCall	Start Question	*M* = .095 (.085)<br>*SD* = .110 (.074)	*M* = .128<br>*SD* = .085	*M* = 3.63<br>*SD* = 1.47
	End Question	*M* = .102 (.084)<br>*SD* = .152 (.099)	*M* = .128<br>*SD* = .098	*M* = 3.24<br>*SD* = 1.47
	Turn-taking	*M* = .029 (.017)<br>*SD* = .071 (.021)	*M* = .071<br>*SD* = .075	*M* = 4.10<br>*SD* = 1.54
	Start Answer	*M* = .039 (.024)<br>*SD* = .108 (.077)	*M* = .022<br>*SD* = .039	*M* = 6.23<br>*SD* = 1.36
	End Answer	*M* = .053 (.041)<br>*SD* = .099 (.063)	*M* = .080<br>*SD* = .069	*M* = 5.33<br>*SD* = 1.53
Real	Start Question	*M* = .054 (.053)<br>*SD* = .060 (.060)	*M* = .114<br>*SD* = .087	*M* = 3.97<br>*SD* = 1.64
	End Question	*M* = .087 (.086)<br>*SD* = .108 (.109)	*M* = .233<br>*SD* = .186	*M* = 3.46<br>*SD* = 1.60
	Turn-taking	*M* = .024 (.023)<br>*SD* = .033 (.034)	*M* = .128<br>*SD* = .151	*M* = 4.01<br>*SD* = 1.62
	Start Answer	*M* = .021 (.021)<br>*SD* = .050 (.050)	*M* = .043<br>*SD* = .091	*M* = 6.13<br>*SD* = 1.79
	End Answer	*M* = .023 (.019)<br>*SD* = .054 (.051)	*M* = .042<br>*SD* = .064	*M* = 5.71<br>*SD* = 1.92

[a]Values after removal of the outlier are in brackets.

Table S1-4. Results for time-course analyses in Pilot Study

		**Prop. gaze to Eye[a]**	**Prop. gaze to Mouth**	**Number facial AUs**
**Condition**	main effect	$F(2,54) = 6.82$<br>$p < .01^{**}$<br>$\eta_p^2 = .202$	$F(2,54) = 4.17$<br>$p < .05^{*}$<br>$\eta_p^2 = .134$	$F(2,54) = 4.63$<br>$p < .05^{*}$<br>$\eta_p^2 = .146$
	V vs. C	$p > .05$ ($p < .05^{*}$)	$p < .01^{**}$	$p > .05$
	V vs. R	$p < .01^{**}$	$p > .05$	$p < .05^{*}$
	C vs. R	$p > .05$	$p > .05$	$p > .05$
**Time-window**	main effect	$F(4,108) = 21.9$<br>$p < .001^{***}$<br>$\eta_p^2 = .447$	$F(4,108) = 44.2$<br>$p < .001^{***}$<br>$\eta_p^2 = .621$	$F(4,108) = 110.2$<br>$p < .001^{***}$<br>$\eta_p^2 = .803$
	Q1 vs. Q2	$p > .05$	$p < .001^{***}$	$p < .001^{***}$
	Q1 vs. TT	$p < .001^{***}$	$p > .05$	$p > .05$
	Q1 vs. A1	$p < .001^{***}$	$p < .001^{***}$	$p < .001^{***}$
	Q1 vs. A2	$p < .001^{***}$	$p < .001^{***}$	$p < .001^{***}$
	Q2 vs. TT	$p < .001^{***}$	$p < .001^{***}$	$p < .001^{***}$
	Q2 vs. A1	$p < .001^{***}$	$p < .001^{***}$	$p < .001^{***}$
	Q2 vs. A2	$p < .01^{**}$	$p < .001^{***}$	$p < .001^{***}$
	TT vs. A1	$p > .05$	$p < .001^{***}$	$p < .001^{***}$
	TT vs. A2	$p > .05$	$p > .05$	$p < .001^{***}$
	A1 vs. A2	$p < .01^{**}$	$p < .05^{*}$	$p < .001^{***}$
**Condition X Time-window**	interaction effect	$F(8,216) = 4.81$<br>$p < .01^{**}$<br>$\eta_p^2 = .151$	$F(8,216) = 3.52$<br>$p < .05^{*}$<br>$\eta_p^2 = .115$	$F(8,216) = 2.60$<br>$p > .05$<br>$\eta_p^2 = .088$
	Q1: V vs. C<br>Q1: V vs. R<br>Q1: C vs. R	$p < .01^{**}$<br>$p < .001^{***}$<br>$p > .05$	$p < .01^{**}$<br>$p < .01^{**}$<br>$p > .05$	-
	Q2: V vs. C<br>Q2: V vs. R<br>Q2: C vs. R	$p > .05$ ($p < .05^{*}$)<br>$p < .05^{*}$ ($p > .05$)<br>$p > .05$	$p < .05^{*}$<br>$p > .05$<br>$p > .05$	-
	TT: V vs. C<br>TT: V vs. R<br>TT: C vs. R	$p > .05$ ($p < .05^{*}$)<br>$p < .05^{*}$ ($p > .05$)<br>$p > .05$	$p < .001^{***}$<br>$p > .05$<br>$p > .05$	-
	A1: V vs. C<br>A1: V vs. R	$p > .05$<br>$p > .05$	$p > .05$<br>$p > .05$	-

A1: C vs. R	$p > .05$	$p > .05$	
A2: V vs. C	$p < .05^{*}$	$p < .05^{*}$	
A2: V vs. R	$p < .01^{**}$	$p < .01^{**}$	-
A2: C vs. R	$p < .05^{*}$	$p > .05$	
V: Q1 vs. Q2	$p > .05$	$p < .05^{*}$	
V: Q1 vs. TT	$p < .001^{***}$	$p < .05^{*}$	
V: Q1 vs. A1	$p < .001^{***}$	$p < .001^{***}$	
V: Q1 vs. A2	$p < .001^{***}$	$p < .001^{***}$	
V: Q2 vs. TT	$p < .001^{***}$	$p < .001^{***}$	-
V: Q2 vs. A1	$p < .001^{***}$	$p < .001^{***}$	
V: Q2 vs. A2	$p < .05^{*}$	$p < .001^{***}$	
V: TT vs. A1	$p > .05$	$p < .001^{***}$	
V: TT vs. A2	$p > .05$	$p < .05^{*}$	
V: A1 vs. A2	$p < .001^{***}$	$p < .001^{***}$	
C: Q1 vs. Q2	$p > .05$	$p < .01^{**}$	
C: Q1 vs. TT	$p < .001^{***}$	$p < .01^{**}$	
C: Q1 vs. A1	$p < .01^{**}$	$p < .001^{***}$	
C: Q1 vs. A2	$p < .01^{**}$	$p < .001^{***}$	
C: Q2 vs. TT	$p < .01^{**}$	$p < .001^{***}$	-
C: Q2 vs. A1	$p < .01^{**}$	$p < .001^{***}$	
C: Q2 vs. A2	$p < .01^{**}$	$p < .001^{***}$	
C: TT vs. A1	$p > .05$	$p < .01^{**}$	
C: TT vs. A2	$p < .05^{*}$	$p > .05$	
C: A1 vs. A2	$p > .05$	$p < .01^{**}$	
R: Q1 vs. Q2	$p < .05^{*}$	$p < .001^{***}$	
R: Q1 vs. TT	$p < .01^{**}$	$p > .05$	
R: Q1 vs. A1	$p < .05^{*}$	$p < .01^{**}$	
R: Q1 vs. A2	$p < .05^{*}$	$p < .001^{***}$	
R: Q2 vs. TT	$p < .01^{**}$	$p < .001^{***}$	-
R: Q2 vs. A1	$p < .01^{**}$	$p < .001^{***}$	
R: Q2 vs. A2	$p < .01^{**}$	$p < .001^{***}$	
R: TT vs. A1	$p > .05$	$p < .001^{***}$	
R: TT vs. A2	$p > .05$	$p < .01^{**}$	
R: A1 vs. A2	$p > .05$	$p > .05$	

[a]Values after removal of the outlier are in brackets. V = Video; C = VideoCall; R = Real; Q1 = start Question; Q2 = end Question; TT = Turn-taking; A1 = start Answer; A2 = end Answer. Asterisks signify difference at $p < .05$ (*), $p < .01$ (**) and $p < .001$ (***).

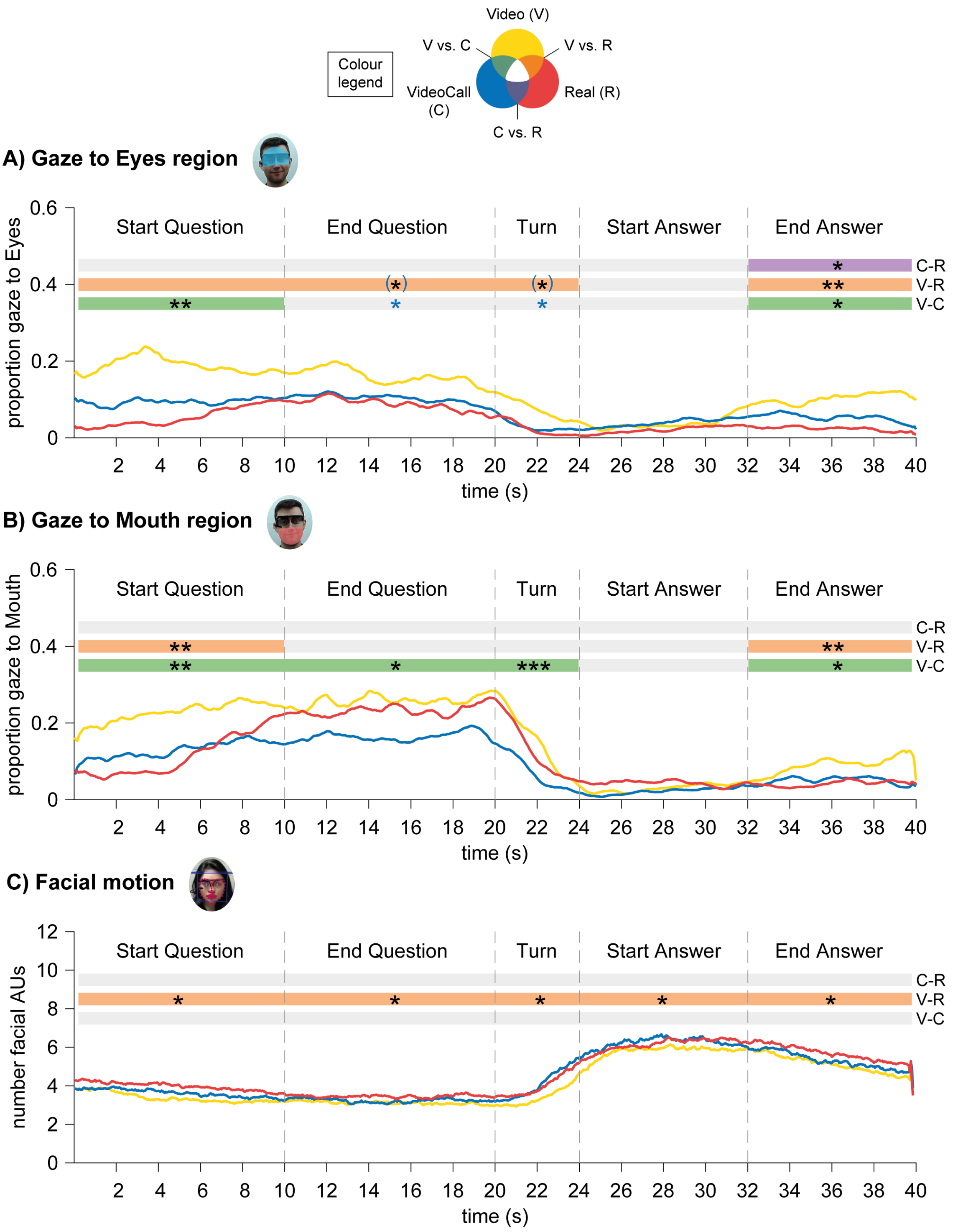

Figure S1-2. Results for time-course analyses in Pilot Study. A) Time-course for proportion of eye gaze directed to Eyes region. B) Time-course for proportion of eye gaze directed to Mouth region. C) Time-course for number of facial AUs. Asterisks signify difference at $p < .05$ (*), $p < .01$ (**) and $p < .001$ (***). Blue asterisks signify difference between V-C after removing the outlier, and blue brackets indicate null difference between V-R after removing the outlier.

Interim discussion

The Pilot Study aimed to investigate how gaze patterns and facial displays are modulated by the belief in being watched and the potential to show true gaze direction in typical individuals. Importantly, post-test questionnaire ratings showed our manipulation across the three conditions was successful: participants perceived the confederate was most reciprocal in the Real condition, and least reciprocal in the Video condition. Note that participants perceived the confederate was more natural in the VideoCall than Video condition, although there were no differences with the Real condition: these inconsistent ratings could be because the task was a structured Q&A interaction, which already lacked the continuity of natural conversation.

Our results show that participants looked less to the eyes of the confederate in the Real and VideoCall conditions compared to the Video condition. Similarly, participants looked less to the mouth of the confederate in the VideoCall compared to the Video condition, although there were no differences between Real and Video conditions. This is in line with previous studies indicating that gaze patterns are modulated by the belief in being watched: participants gaze less to a live partner compared to a video-clip of the same partner, because in a live interaction eye gaze has a dual function of both perceiving and signalling information (Cañigueral & Hamilton, 2019a; Gobel et al., 2015; Laidlaw et al., 2011).

A core question is how participants use eye gaze in relation to other social signals (i.e. speech) during live communicative exchanges. This can give further insight into how we plan eye movements to maximise the information we gather from others and optimise the information we send to others. To study detailed dynamics of eye gaze, we looked at differences between conditions in 5 different time-windows along the trial time-course:

start of the question, end of the question, turn-taking, start of the answer, and end of the answer. In line with previous studies (Hessels et al., 2019; Ho et al., 2015; Kendon, 1967), we found that participants generally looked more to the eyes and mouth of the confederate during the Question phases than during the Answer phases. However, we found that this pattern was also true for the Video condition, where participants were aware they were not in a live conversation, and so there was no need to regulate turn-taking. This opens up an interesting question for future research: do we look away from faces when speaking to signal we want to keep the turn, as suggested by the regulatory function of gaze (Kendon, 1967), or rather because looking at faces is cognitively demanding and might otherwise interfere with speaking (Beattie, 1981; Glenberg, Schroeder, & Robertson, 1998; Kendon, 1967; Markson & Paterson, 2009)? The fact that we find the same modulation across all three conditions indicates that the latter might be the case.

As expected, differences between the Real condition and the VideoCall or Video conditions happened either at the start of the interaction (start Question) or at the end of the interaction (end Answer). Based on previous studies, we predicted that participants would look more to the Real confederate at the start of the Question to signal interest in what she was saying (Argyle & Cook, 1976; Kendon, 1967), and at the end of the Answer to signal that they were ending the answer (Ho et al., 2015; Kendon, 1967) or to monitor facial displays of the confederate (Efran, 1968; Efran & Broughton, 1966; Kendon, 1967; Kleinke, 1986). However, at these time-windows participants looked *less* to the eyes and mouth of the confederate in the Real condition. This suggests that participants were averting gaze to reduce the intensity of the interaction or arousal associated with live eye contact with a stranger (Argyle & Dean, 1965; Kendon, 1967; Pönkänen et al., 2011). Note that we did not find any differences during Turn-taking. As we discuss below, this could be because triggers

for turn-taking in the Real and VideoCall conditions (where the speech speed of the confederate slightly varied among questions and participants) were not as accurate as in the Video condition (which uses pre-recorded videos).

Comparison between the VideoCall and Video conditions showed that there was a similar pattern of results: participants looked less to the eyes and mouth of the confederate in the VideoCall condition, both at the start and end of the interaction. The similar patterns between Real and VideoCall conditions indicates that our manipulation of true gaze direction did not have strong effects on gaze planning. However, in the VideoCall condition participants also looked less to the confederate during the end of the Question phase and Turn-taking. Unfortunately, as mentioned earlier, our interpretation of these findings is constrained by the fact that triggers for turn-taking in the VideoCall condition were not as accurate as in the Video condition.

Finally, we also looked at patterns of facial motion. Results showed that participants moved their face more in the Real than in the Video condition (although there were no differences between VideoCall and Video conditions). This is in line with previous studies showing that participants make more facial displays when being watched, which suggests that we use facial displays as a social signal (Chovil, 1991b; Crivelli & Fridlund, 2018; Fridlund, 1991; Hietanen et al., 2018). When looking at the time-course of facial motion along the trial we found that, as expected, participants moved their face more during the Answer phase than during the Question phase. Although we hypothesised that participants would do this to help communicate their spoken answers in the Real and VideoCall conditions (Chovil, 1991a), we found that this pattern was also true for the Video condition. This could be because the face-tracking algorithm was also picking up facial motion related to moving the mouth for speech production. We discuss this limitation below.

There were two main limitations to the Pilot Study. First, facial motion effects might be confounded with effects related to speech production, since moving the mouth to speak is likely to be detected as facial motion. Second, the triggers for turn-taking were not as accurate in the VideoCall and Real conditions as in the Video condition, since the speech speed of the confederate slightly varied among questions and participants. To address these limitations, in the Autism Study we recorded the voice of participants and confederate with two lapel microphones. The participants' microphone allowed us to detect when participants were speaking and account for this in the facial motion analysis. The confederate's microphone was used as a trigger system, where the confederate was instructed to tap on the microphone at the end of each question: this allowed us to automatically detect a peak in the audio signal that accurately corresponded to the time of turn-taking.

Building on our findings from the Pilot Study, in the Autism Study we tested a matched sample of typical and autistic participants. Previous studies suggest that autistic individuals have difficulties in appropriately using eye gaze during social interactions, but evidence is mixed (Falck-Ytter & Von Hofsten, 2011). Thus, in the Autism Study we examined differences between typical and autistic patterns of eye gaze and facial displays in a communicative situation.

S2. List of questions

Autism Study

Set 1 - Video

1) Summer is coming and you are planning your holidays. Would you rather: option A, take a European sight-seeing vacation, or Option B, take a relaxing Caribbean vacation?
2) You have saved £150. Would you rather: option A, give it to a homeless shelter, or Option B, spend it on a weekend trip?
3) Your vision skills will be modified for a day. Would you rather: option A, only see infrared rays, or Option B, only see ultraviolet rays?
4) You have some spare mornings this year. Would you rather: option A, work as an assistant in a company, or Option B, volunteer in a nursing home?
5) You want to be very skilled at something. Would you rather: option A, be able to play all musical instruments, or Option B, be able to speak all foreign languages?
6) Your boss gives you a day off. Would you rather: option A, spend the day hiking, or Option B, spend the day cycling?
7) You have a free afternoon this weekend. Would you rather: option A, collaborate in a charity event, or Option B, do an outdoors activity you like?
8) You have a free afternoon. Would you rather: option A, go to a museum, or Option B, go to the theatre?
9) You have saved £280. Would you rather: option A, buy something you really want, or Option B, donate it to a fundraising event?
10) You are very impatient and don’t like to wait. Would you rather: option A, never have to wait in line at airports, or Option B, never have to wait in line at stores?

<u>Set 2 - VideoCall</u>

1) You don't have much work this year. Would you rather: option A, work part-time in a department store, or Option B, volunteer in a local charity shop?
2) You have won a voucher. Would you rather: option A, have free coffee for a year, or Option B, have free cake for a year?
3) A witch will make your wish come true. Would you rather: option A, find your true love, or Option B, find £100,000?
4) You have saved £300. Would you rather: option A, donate it to an international NGO, or Option B, buy new furniture for your place?
5) You have a free weekend. Would you rather: option A, spend it by the sea, or Option B, spend it in the mountains?
6) You have a free day this week. Would you rather: option A, participate in a fundraising campaign, or Option B, enjoy a relaxing day to do things you like?
7) You are going to the cinema this evening. Would you rather: option A, watch a fantasy film, or Option B, watch a comedy film?
8) Your watch is broken. Would you rather: option A, always be 10 minutes late, or Option B, always be 20 minutes early?
9) You have a special power during this month. Would you rather: option A, discover a new planet, or Option B, discover a new animal specie?
10) You have saved £210. Would you rather: option A, spend it during your holidays, or Option B, give it to a local charity?

<u>Set 3 - Real</u>

1) You have some spare time this year. Would you rather: option A, volunteer in a homeless shelter, or Option B, find a part-time job in a café?

2) You have a free evening. Would you rather: option A, spend it in a concert, or Option B, spend it at the cinema?
3) You have saved £180. Would you rather: option A, donate it to a charity, or Option B, spend it on a trip?
4) You want to reduce your electricity expenses for a week. Would you rather: option A, live without the Internet, or Option B, live without heating and hot water?
5) It's Christmas and you are about to open your presents. Would you rather: option A, receive cash, or Option B, receive a gift?
6) You have saved £320. Would you rather: option A, do some refurbishments in your home, or Option B, give it to an online fundraising cause?
7) You don't have access to water for a day. Would you rather: option A, only drink coke, or Option B, only drink juice?
8) You have the special power to become an animal for a day. Would you rather: option A, become a fish, or Option B, become a bird?
9) You have a free evening during the week. Would you rather: option A, have a nice and relaxing dinner, or Option B, volunteer in a soup kitchen?
10) A wizard will give you a superpower for a day. Would you rather: option A, be invisible, or Option B, be able to fly?

Pilot Study

Set 1 - Video

1) Summer is coming and you are planning your holidays. Would you rather: option A, take a European sight-seeing vacation, or option B, take a relaxing Caribbean vacation?
2) You have saved £200 during the last month. Would you rather: option A, donate it to a local fundraising event, or option B, spend it on a trip?

3) You have some spare time this term. Would you rather: option A, go to the gym, or option B, volunteer for a children's sport charity?
4) A witch casts a spell on you and lets you choose. Would you rather: option A, go on your perfect holiday for a month, or option B, volunteer in a developing country for a month?
5) Your vision skills will be modified for a day. Would you rather: option A, only see infrared rays, or option B, only see ultraviolet rays?
6) You have a free afternoon this weekend. Would you rather: option A, help prepare games for a charity event, or option B, spend the afternoon in a café with your friends?
7) Your boss gives you a day off. Would you rather: option A, spend the day hiking, or option B, spend the day cycling?
8) A magician gives you £400. Would you rather: option A, give it to a disabilities-related charity, or option B, spend it on your next holiday?
9) You have some spare mornings this year. Would you rather: option A, work as an assistant in your department, or option B, volunteer in a nursing home?
10) You have a lot of work today. Would you rather: option A, help a colleague who is stuck in his project, or option B, concentrate and finish your work quickly?
11) You want to be very skilled at something. Would you rather: option A, be able to play all musical instruments, or option B, be able to speak all foreign languages?
12) You have found a £20 note in the street. Would you rather: option A, give it to a homeless busker, or option B, save it for a concert of your favourite music band?

<u>Set 2 - VideoCall</u>

1) You don't have much work this month. Would you rather: option A, do some personal tutoring with children, or option B, volunteer in a children-related charity?

2) You have some spare time this term. Would you rather: option A, volunteer teaching disabled people how to play an instrument, or option B, learn to play an instrument?
3) A witch casts a spell on you and lets you choose. Would you rather: option A, provide resources to developing countries for a year, or option B, travel around the world for a year?
4) You have found a £10 note in the staircase of your building. Would you rather: option A, save it to have a treat next weekend, or option B, give it to a homeless man in your neighbourhood?
5) You are free on a Saturday. Would you rather: option A, help organise a game for a fundraising event, or option B, spend the day out with your friends?
6) You have won a voucher. Would you rather: option A, have free coffee for a year, or option B, have free cake for a year?
7) After Christmas you have saved £200. Would you rather: option A, buy new furniture for your place, or option B, give it to a charity for old people?
8) A witch will make your wish come true. Would you rather: option A, find your true love, or option B, find £100,000?
9) A magician gives you £400. Would you rather: option A, spend it on a nice holiday next summer, or option B, donate it to help people in poor countries?
10) You have a free weekend to spend with your friends. Would you rather: option A, spend it by the sea, or option B, spend it in the mountains?
11) You are going to the cinema this evening. Would you rather: option A, watch a fantasy film, or option B, watch a comedy film?
12) You have quite a lot of homework to do. Would you rather: option A, help a classmate who struggles with homework, or option B, finish your homework as soon as possible?

Set 3 - Real

1) You have some spare time this year. Would you rather: option A, volunteer in a homeless shelter, or option B, find a part-time job in a café?
2) You have a free evening. Would you rather: option A, spend it in a concert, or option B, spend it at the cinema?
3) You have won £500 in the lottery. Would you rather: option A, donate it to help developing countries, or option B, buy something you really want?
4) You want to reduce your electricity expenses for a week. Would you rather: option A, live without the Internet, or option B, live without heating and hot water?
5) You have one free evening during the week. Would you rather: option A, have a nice dinner with your flatmates, or option B, volunteer in a charity that helps old people?
6) A wizard gives you £100. Would you rather: option A, give it to an NGO that helps protect natural environments, or option B, spend it on a nice weekend out of town?
7) You don't have access to water for a day. Would you rather: option A, only drink coke, or option B, only drink tea?
8) A magician casts a spell on you and lets you choose. Would you rather: option A, have no work for a week, or option B, help disabled people for a week?
9) You have found a £20 note in the changing room of a shop. Would you rather: option A, buy a new jumper you need, or option B, give it to a homeless woman?
10) You are in the airport and need to pass the security check. Would you rather: option A, let a big group of old people go ahead, or option B, hurry up to have the place in the fastest line?
11) A wizard will give you a superpower for a day. Would you rather: option A, be invisible, or option B, be able to fly?

12) You are taking the mornings off work this week. Would you rather: option A, do some leisure activity, or option B, collaborate in a fundraising event?

S3. Post-test questionnaire

Section 1

I think the interaction in the video was very natural.

(disagree) 0 1 2 3 4 5 6 7 8 (agree)

I think the interaction in the video was very reciprocal.

(disagree) 0 1 2 3 4 5 6 7 8 (agree)

I think the interaction in the video-conference was very natural.

(disagree) 0 1 2 3 4 5 6 7 8 (agree)

I think the interaction in the video-conference was very reciprocal.

(disagree) 0 1 2 3 4 5 6 7 8 (agree)

I think the interaction face-to-face was very natural.

(disagree) 0 1 2 3 4 5 6 7 8 (agree)

I think the interaction face-to-face was very reciprocal.

(disagree) 0 1 2 3 4 5 6 7 8 (agree)

Section 2

Which interaction (video / video-conference / face-to-face) did you like the most? Why?

Which interaction (video / video-conference / face-to-face) did you like the least? Why?

What do you think was the purpose of the experiment?

S4. Speech analysis

We computed the mean proportion of speech for each Condition, across all time-points and trials. We then performed a 2-way repeated measures ANOVA with mean proportion of speech as dependent variable, Condition (Video, VideoCall, Real) as within-subject factor, and Group (Typical, Autism) as between-subject factor. See Table S4-1 for descriptives (mean and SD) on proportion of speech. There was no main effect of Condition, $F(2,100) = 1.18$, $p > .05$, $\eta_p^2 = .023$, no main effect of Group, $F(1,50) = .206$, $p > .05$, $\eta_p^2 = .004$, and no interaction effect between Condition and Group, $F(2,100) = 1.04$, $p > .05$, $\eta_p^2 = .020$ (Figure S4-1).

Table S4-1. Descriptives for aggregated analysis of speech in Autism Study

**Condition**	Video		VideoCall		Real	
**Group**	Typical	Autism	Typical	Autism	Typical	Autism
**Proportion of speech**	*M* = .118<br>*SD* = .043	*M* = .120<br>*SD* = .050	*M* = .118<br>*SD* = .046	*M* = .131<br>*SD* = .051	*M* = .117<br>*SD* = .051	*M* = .120<br>*SD* = .049

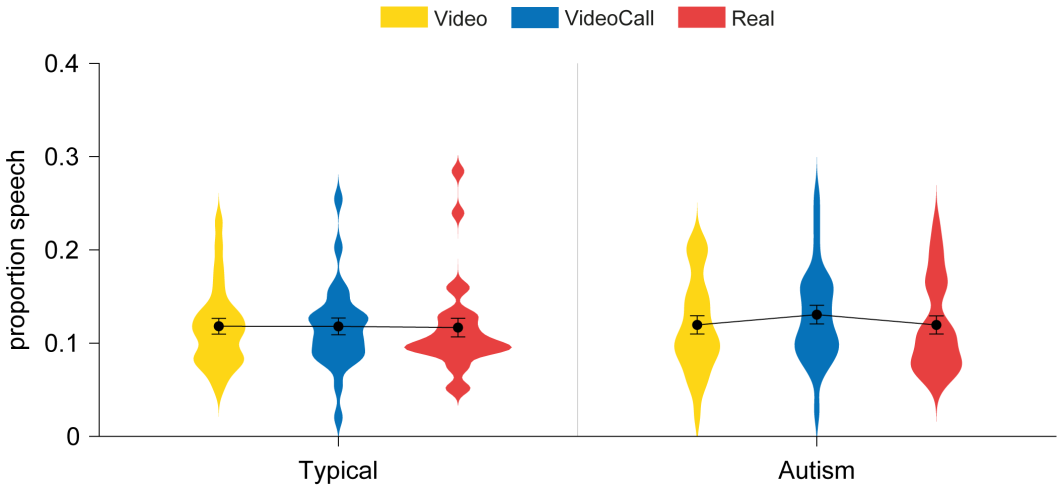

Figure S4-1. Results for speech analysis in the Autism Study. Proportion of speech for each Condition and Group. Mean (filled circle), SE (error bars), and frequency of values (width of distribution).

Table S1. Results for time-course analyses in Autism Study: eye gaze

		**Prop. gaze to Eye**	**Prop. gaze to Mouth**
**Condition**	main effect	$F(2,100) = 10.4$<br>$p < .001^{***}$<br>$n_p^2 = .172$	$F(2,100) = 4.83$<br>$p < .05^{*}$<br>$n_p^2 = .088$
	V vs. C	$p < .01^{**}$	$p < .05^{*}$
	V vs. R	$p < .01^{**}$	$p > .05$
	C vs. R	$p > .05$	$p > .05$
**Time-window**	main effect	$F(4,200) = 39.7$<br>$p < .001^{***}$<br>$n_p^2 = .443$	$F(4,200) = 38.7$<br>$p < .001^{***}$<br>$n_p^2 = .437$
	Q1 vs. Q2	$p < .001^{***}$	$p < .001^{***}$
	Q1 vs. TT	$p < .05^{*}$	$p < .001^{***}$
	Q1 vs. A1	$p < .01^{**}$	$p < .05^{*}$
	Q1 vs. A2	$p < .01^{**}$	$p < .01^{**}$
	Q2 vs. TT	$p < .01^{**}$	$p > .05$
	Q2 vs. A1	$p < .001^{***}$	$p < .001^{***}$
	Q2 vs. A2	$p < .001^{***}$	$p < .001^{***}$
	TT vs. A1	$p < .001^{***}$	$p < .001^{***}$
	TT vs. A2	$p < .001^{***}$	$p < .001^{***}$
	A1 vs. A2	$p > .05$	$p > .05$
**Group**	main effect	$F(1,50) = .214$<br>$p > .05$<br>$n_p^2 = .004$	$F(1,50) = 2.08$<br>$p > .05$<br>$n_p^2 = .040$
**Condition X Time-window**	interaction effect	$F(8,400) = 5.55$<br>$p < .001^{***}$<br>$n_p^2 = .100$	$F(8,400) = 4.86$<br>$p < .01^{**}$<br>$n_p^2 = .089$
	Q1: V vs. C	$p < .001^{***}$	$p > .05$
	Q1: V vs. R	$p < .001^{***}$	$p < .001^{***}$
	Q1: C vs. R	$p < .05^{*}$	$p < .01^{**}$
	Q2: V vs. C	$p < .01^{**}$	$p < .05^{*}$
	Q2: V vs. R	$p > .05$	$p > .05$
	Q2: C vs. R	$p > .05$	$p > .05$
	TT: V vs. C	$p < .01^{**}$	$p < .01^{**}$
	TT: V vs. R	$p < .01^{**}$	$p < .05^{*}$

TT: C vs. R	$p > .05$	$p > .05$
A1: V vs. C	$p < .01^{**}$	$p < .05^{*}$
A1: V vs. R	$p > .05$	$p < .05^{*}$
A1: C vs. R	$p > .05$	$p > .05$
A2: V vs. C	$p < .05^{*}$	$p > .05$
A2: V vs. R	$p < .05^{*}$	$p > .05$
A2: C vs. R	$p > .05$	$p > .05$
V: Q1 vs. Q2	$p < .001^{***}$	$p < .001^{***}$
V: Q1 vs. TT	$p < .05^{*}$	$p < .01^{**}$
V: Q1 vs. A1	$p < .001^{***}$	$p < .01^{**}$
V: Q1 vs. A2	$p < .001^{***}$	$p < .001^{***}$
V: Q2 vs. TT	$p < .01^{**}$	$p > .05$
V: Q2 vs. A1	$p < .001^{***}$	$p < .001^{***}$
V: Q2 vs. A2	$p < .001^{***}$	$p < .001^{***}$
V: TT vs. A1	$p < .001^{***}$	$p < .001^{***}$
V: TT vs. A2	$p < .001^{***}$	$p < .001^{***}$
V: A1 vs. A2	$p < .05^{*}$	$p > .05$
C: Q1 vs. Q2	$p < .001^{***}$	$p < .001^{***}$
C: Q1 vs. TT	$p > .05$	$p > .05$
C: Q1 vs. A1	$p < .01^{**}$	$p < .01^{**}$
C: Q1 vs. A2	$p < .01^{**}$	$p < .01^{**}$
C: Q2 vs. TT	$p < .05^{*}$	$p < .01^{**}$
C: Q2 vs. A1	$p < .001^{***}$	$p < .001^{***}$
C: Q2 vs. A2	$p < .001^{***}$	$p < .001^{***}$
C: TT vs. A1	$p < .001^{***}$	$p < .001^{***}$
C: TT vs. A2	$p < .01^{**}$	$p < .01^{**}$
C: A1 vs. A2	$p > .05$	$p > .05$
R: Q1 vs. Q2	$p < .05^{*}$	$p < .001^{***}$
R: Q1 vs. TT	$p < .001^{***}$	$p < .001^{***}$
R: Q1 vs. A1	$p > .05$	$p > .05$
R: Q1 vs. A2	$p > .05$	$p > .05$
R: Q2 vs. TT	$p < .01^{**}$	$p < .01^{**}$
R: Q2 vs. A1	$p < .001^{***}$	$p < .001^{***}$
R: Q2 vs. A2	$p < .001^{***}$	$p < .001^{***}$
R: TT vs. A1	$p < .001^{***}$	$p < .001^{***}$

	R: TT vs. A2	$p < .01^{**}$	$p < .001^{***}$
	R: A1 vs. A2	$p > .05$	$p < .01^{**}$
**Condition X Group**	interaction effect	$F(2,100) = 2.10$<br>$p > .05$<br>$n_p^2 = .040$	$F(2,100) = .168$<br>$p > .05$<br>$n_p^2 = .003$
**Time-window X Group**	interaction effect	$F(4,200) = .783$<br>$p > .05$<br>$n_p^2 = .015$	$F(4,200) = .964$<br>$p > .05$<br>$n_p^2 = .019$
**Condition X Time-window X Group**	interaction effect	$F(8,400) = 2.81$<br>$p < .05^{*}$<br>$n_p^2 = .053$	$F(8,400) = .553$<br>$p > .05$<br>$n_p^2 = .011$
	R,Q1: Typ vs. Aut	$p < .05^{*}$	-
	All other contrasts between Typ and Aut are not significant		
	Typ,Q1: V vs. C	$p < .05^{*}$	-
	Typ,Q1: V vs. R	$p < .001^{***}$	
	Typ,Q1: C vs. R	$p < .01^{**}$	
	Typ,Q2: V vs. C	$p > .05$	-
	Typ,Q2: V vs. R	$p > .05$	
	Typ,Q2: C vs. R	$p > .05$	
	Typ,TT: V vs. C	$p > .05$	-
	Typ,TT: V vs. R	$p > .05$	
	Typ,TT: C vs. R	$p > .05$	
	Typ,A1: V vs. C	$p > .05$	-
	Typ,A1: V vs. R	$p > .05$	
	Typ,A1: C vs. R	$p > .05$	
	Typ,A2: V vs. C	$p < .05^{*}$	-
	Typ,A2: V vs. R	$p > .05$	
	Typ,A2: C vs. R	$p > .05$	
	Aut,Q1: V vs. C	$p < .01^{**}$	-
	Aut,Q1: V vs. R	$p < .01^{**}$	
	Aut,Q1: C vs. R	$p > .05$	
	Aut,Q2: V vs. C	$p < .01^{**}$	-
	Aut,Q2: V vs. R	$p > .05$	
	Aut,Q2: C vs. R	$p < .01^{**}$	
	Aut,TT: V vs. C	$p < .001^{***}$	-

Aut,TT: V vs. R	$p < .05^{*}$	
Aut,TT: C vs. R	$p < .05^{*}$	
Aut,A1: V vs. C	$p < .01^{**}$	
Aut,A1: V vs. R	$p > .05$	-
Aut,A1: C vs. R	$p > .05$	
Aut,A2: V vs. C	$p < .01^{**}$	
Aut,A2: V vs. R	$p > .05$	-
Aut,A2: C vs. R	$p > .05$	
Typ,V: Q1 vs. Q2	$p < .05^{*}$	
Typ,V: Q1 vs. TT	$p > .05$	
Typ,V: Q1 vs. A1	$p < .001^{***}$	
Typ,V: Q1 vs. A2	$p < .001^{***}$	
Typ,V: Q2 vs. TT	$p < .001^{***}$	
Typ,V: Q2 vs. A1	$p < .001^{***}$	-
Typ,V: Q2 vs. A2	$p < .001^{***}$	
Typ,V: TT vs. A1	$p < .01^{**}$	
Typ,V: TT vs. A2	$p < .05^{*}$	
Typ,V: A1 vs. A2	$p < .05^{*}$	
Typ,C: Q1 vs. Q2	$p < .001^{***}$	
Typ,C: Q1 vs. TT	$p > .05$	
Typ,C: Q1 vs. A1	$p < .05^{*}$	
Typ,C: Q1 vs. A2	$p < .05^{*}$	
Typ,C: Q2 vs. TT	$p < .05^{*}$	
Typ,C: Q2 vs. A1	$p < .001^{***}$	-
Typ,C: Q2 vs. A2	$p < .001^{***}$	
Typ,C: TT vs. A1	$p < .001^{***}$	
Typ,C: TT vs. A2	$p < .01^{**}$	
Typ,C: A1 vs. A2	$p > .05$	
Typ,R: Q1 vs. Q2	$p < .001^{***}$	
Typ,R: Q1 vs. TT	$p < .05^{*}$	
Typ,R: Q1 vs. A1	$p > .05$	
Typ,R: Q1 vs. A2	$p > .05$	-
Typ,R: Q2 vs. TT	$p < .05^{*}$	
Typ,R: Q2 vs. A1	$p < .01^{**}$	
Typ,R: Q2 vs. A2	$p < .01^{**}$	

Typ,R: TT vs. A1	$p > .05$	
Typ,R: TT vs. A2	$p > .05$	
Typ,R: A1 vs. A2	$p > .05$	
Aut,V: Q1 vs. Q2	$p < .001^{***}$	-
Aut,V: Q1 vs. TT	$p < .001^{***}$	
Aut,V: Q1 vs. A1	$p < .001^{***}$	
Aut,V: Q1 vs. A2	$p < .01^{**}$	
Aut,V: Q2 vs. TT	$p > .05$	
Aut,V: Q2 vs. A1	$p < .001^{***}$	
Aut,V: Q2 vs. A2	$p < .001^{***}$	
Aut,V: TT vs. A1	$p < .001^{***}$	
Aut,V: TT vs. A2	$p < .001^{***}$	
Aut,V: A1 vs. A2	$p > .05$	
Aut,C: Q1 vs. Q2	$p > .05$	-
Aut,C: Q1 vs. TT	$p > .05$	
Aut,C: Q1 vs. A1	$p > .05$	
Aut,C: Q1 vs. A2	$p > .05$	
Aut,C: Q2 vs. TT	$p > .05$	
Aut,C: Q2 vs. A1	$p < .05^{*}$	
Aut,C: Q2 vs. A2	$p < .05^{*}$	
Aut,C: TT vs. A1	$p < .05^{*}$	
Aut,C: TT vs. A2	$p > .05$	
Aut,C: A1 vs. A2	$p > .05$	
Aut,R: Q1 vs. Q2	$p < .001^{***}$	-
Aut,R: Q1 vs. TT	$p < .01^{**}$	
Aut,R: Q1 vs. A1	$p > .05$	
Aut,R: Q1 vs. A2	$p > .05$	
Aut,R: Q2 vs. TT	$p < .05^{*}$	
Aut,R: Q2 vs. A1	$p < .001^{***}$	
Aut,R: Q2 vs. A2	$p < .001^{***}$	
Aut,R: TT vs. A1	$p < .001^{***}$	
Aut,R: TT vs. A2	$p < .01^{**}$	
Aut,R: A1 vs. A2	$p > .05$	

V = Video; C = VideoCall; R = Real; Q1 = start Question; Q2 = end Question; TT = Turn-taking; A1 = start Answer; A2 = end Answer; Typ = typical; Aut = autism. Asterisks signify difference at $p < .05$ (*), $p < .01$ (**) and $p < .001$ (***).

Table S2. Results for time-course analyses in Autism Study: facial motion

		Number facial AUs
Condition	main effect	$F(2,7657.5) = 59.0$ $p < .001^{***}$
	V vs. C	$p > .05$
	V vs. R	$p < .05^{*}$
	C vs. R	$p > .05$
Time-window	main effect	$F(4,7669.2) = 76.0$ $p < .001^{***}$
	Q1 vs. Q2	$p < .001^{***}$
	Q1 vs. TT	$p > .05$
	Q1 vs. A1	$p < .001^{***}$
	Q1 vs. A2	$p < .001^{***}$
	Q2 vs. TT	$p < .001^{***}$
	Q2 vs. A1	$p < .001^{***}$
	Q2 vs. A2	$p < .001^{***}$
	TT vs. A1	$p < .001^{***}$
	TT vs. A2	$p < .001^{***}$
	A1 vs. A2	$p > .05$
Group	main effect	$F(1,51.003) = 1.48$ $p > .05$
Speech	main effect	$F(1,6506.7) = .233$ $p > .05$
Condition X Time-window	interaction effect	$F(8,7653.5) = 1.99$ $p < .05^{*}$
	Q1: V vs. C	$p < .001^{***}$
	Q1: V vs. R	$p < .01^{**}$
	Q1: C vs. R	$p > .05$
	Q2: V vs. C	$p < .001^{***}$
	Q2: V vs. R	$p < .001^{***}$
	Q2: C vs. R	$p < .05^{*}$
	TT: V vs. C	$p < .001^{***}$
	TT: V vs. R	$p < .001^{***}$
	TT: C vs. R	$p > .05$

A1: V vs. C	$p < .001^{***}$
A1: V vs. R	$p < .001^{***}$
A1: C vs. R	$p > .05$
A2: V vs. C	$p < .05^{*}$
A2: V vs. R	$p < .05^{*}$
A2: C vs. R	$p > .05$
V: Q1 vs. Q2	$p < .001^{***}$
V: Q1 vs. TT	$p > .05$
V: Q1 vs. A1	$p < .001^{***}$
V: Q1 vs. A2	$p < .001^{***}$
V: Q2 vs. TT	$p > .05$
V: Q2 vs. A1	$p < .001^{***}$
V: Q2 vs. A2	$p < .001^{***}$
V: TT vs. A1	$p < .001^{***}$
V: TT vs. A2	$p < .001^{***}$
V: A1 vs. A2	$p < .01^{**}$
C: Q1 vs. Q2	$p < .01^{**}$
C: Q1 vs. TT	$p > .05$
C: Q1 vs. A1	$p < .001^{***}$
C: Q1 vs. A2	$p < .001^{***}$
C: Q2 vs. TT	$p < .001^{***}$
C: Q2 vs. A1	$p < .001^{***}$
C: Q2 vs. A2	$p < .001^{***}$
C: TT vs. A1	$p < .001^{***}$
C: TT vs. A2	$p < .001^{***}$
C: A1 vs. A2	$p > .05$
R: Q1 vs. Q2	$p < .001^{***}$
R: Q1 vs. TT	$p < .05^{*}$
R: Q1 vs. A1	$p < .001^{***}$
R: Q1 vs. A2	$p < .001^{***}$
R: Q2 vs. TT	$p < .001^{***}$
R: Q2 vs. A1	$p < .001^{***}$
R: Q2 vs. A2	$p < .001^{***}$
R: TT vs. A1	$p < .001^{***}$
R: TT vs. A2	$p < .001^{***}$

	R: A1 vs. A2	*p* > .05
**Condition X Group**	interaction effect	*F*(2,7657.5) = .496 *p* > .05
**Time-window X Group**	interaction effect	*F*(4,7669.2) = 1.52 *p* > .05
**Speech X Group**	interaction effect	*F*(2,5617.9) = .092 *p* > .05
**Time-window X Condition**	interaction effect	*F*(8,7653.5) = 1.99 *p* > .05
**Speech X Condition**	interaction effect	*F*(2,7660.8) = 1.86 *p* > .05
**Time-window X Speech**	interaction effect	*F*(3,7676.3) = .883 *p* > .05
**Group X Condition X Time-window**	interaction effect	*F*(8,7653.5) = .875 *p* > .05
**Group X Condition X Speech**	interaction effect	*F*(2,7675.4) = .729 *p* > .05
**Group X Time-window X Speech**	interaction effect	*F*(3,7675.3) = .376 *p* > .05
**Condition X Time-window X Speech**	interaction effect	*F*(5,7669.9) = 1.88 *p* > .05
**Group X Condition X Time-window X Speech**	interaction effect	*F*(4,7673.9) = 1.19 *p* > .05

V = Video; C = VideoCall; R = Real; Q1 = start Question; Q2 = end Question; TT = Turn-taking; A1 = start Answer; A2 = end Answer. Asterisks signify difference at p < .05 (*), p < .01 (**) and p < .001 (***).
